# A prophylactic multivalent vaccine against different filovirus species is immunogenic and provides protection from lethal infections with *Ebolavirus* and *Marburgvirus* species in non-human primates

**DOI:** 10.1371/journal.pone.0192312

**Published:** 2018-02-20

**Authors:** Benoit Callendret, Jort Vellinga, Kerstin Wunderlich, Ariane Rodriguez, Robin Steigerwald, Ulrike Dirmeier, Cedric Cheminay, Ariane Volkmann, Trevor Brasel, Ricardo Carrion, Luis D. Giavedoni, Jean L. Patterson, Chad E. Mire, Thomas W. Geisbert, Jay W. Hooper, Mo Weijtens, Jutta Hartkoorn-Pasma, Jerome Custers, Maria Grazia Pau, Hanneke Schuitemaker, Roland Zahn

**Affiliations:** 1 Janssen Vaccines & Prevention B.V., Leiden, Netherlands; 2 Bavarian Nordic GmbH, Martinsried, Germany; 3 University of Texas Medical Branch, Galveston, Texas, United States of America; 4 Department of Virology and Immunology, Texas Biomedical Research Institute, San Antonio, Texas, United States of America; 5 Virology Division, US Army Medical Research Institute of Infectious Diseases, Fort Detrick, Maryland, United States of America; Division of Clinical Research, UNITED STATES

## Abstract

The search for a universal filovirus vaccine that provides protection against multiple filovirus species has been prompted by sporadic but highly lethal outbreaks of *Ebolavirus* and *Marburgvirus* infections. A good prophylactic vaccine should be able to provide protection to all known filovirus species and as an upside potentially protect from newly emerging virus strains. We investigated the immunogenicity and protection elicited by multivalent vaccines expressing glycoproteins (GP) from Ebola virus (EBOV), Sudan virus (SUDV), Taï Forest virus (TAFV) and Marburg virus (MARV). Immune responses against filovirus GP have been associated with protection from disease. The GP antigens were expressed by adenovirus serotypes 26 and 35 (Ad26 and Ad35) and modified Vaccinia virus Ankara (MVA) vectors, all selected for their strong immunogenicity and good safety profile. Using fully lethal NHP intramuscular challenge models, we assessed different vaccination regimens for immunogenicity and protection from filovirus disease. Heterologous multivalent Ad26-Ad35 prime-boost vaccination regimens could give full protection against MARV (range 75%-100% protection) and EBOV (range 50% to 100%) challenge, and partial protection (75%) against SUDV challenge. Heterologous multivalent Ad26-MVA prime-boost immunization gave full protection against EBOV challenge in a small cohort study. The use of such multivalent vaccines did not show overt immune interference in comparison with monovalent vaccines. Multivalent vaccines induced GP-specific antibody responses and cellular IFNγ responses to each GP expressed by the vaccine, and cross-reactivity to TAFV GP was detected in a trivalent vaccine expressing GP from EBOV, SUDV and MARV. In the EBOV challenge studies, higher humoral EBOV GP-specific immune responses (p = 0.0004) were associated with survival from EBOV challenge and less so for cellular immune responses (p = 0.0320). These results demonstrate that it is feasible to generate a multivalent filovirus vaccine that can protect against lethal infection by multiple members of the filovirus family.

## Introduction

Filoviruses, which include the genera *Ebolavirus* and *Marburgvirus*, cause sporadic outbreaks of severe hemorrhagic disease in humans with case mortality rates between 25% and 90% [1]. Outbreaks of filovirus infection start when humans have direct contact with infected animals or with their contaminated body fluids, spreading in the human population by human-to-human transmission [2]. Mapping models of previous outbreaks and reservoir habitats in Africa have identified a population of 22 million people who are at potential risk from *Ebolavirus* transmission, and 105 million people who are at potential risk from *Marburgvirus* transmission [3, 4]. The lack of specific treatment, high mortality rates, and substantial social and economic impact of the disease indicate the need for vaccines to prevent infection in a cost effective manner.

Vaccination strategies to combat filovirus infection differentiate between prophylactic vaccination and reactive use during outbreaks. Prophylactic vaccination would be beneficial to populations deemed at risk from geographical or occupational exposure and may be administered on a large scale. Due to the unpredictability of outbreaks, an effective prophylactic filovirus vaccine should protect against all potentially circulating filovirus species. At present there are five known species of *Ebolavirus*: *Zaire ebolavirus* (Ebola virus, EBOV), *Sudan ebolavirus* (Sudan virus, SUDV), *Taï Forest ebolavirus* (Taï Forest virus, TAFV), *Reston ebolavirus* (Reston virus, RESTV), and *Bundibugyo ebolavirus* (Bundibugyo virus, BDBV). There are two known viruses in the *Marburg marburgvirus* species: Marburg virus (MARV) and Ravn virus (RAVV). In addition, *Cuevavirus* has a single species, *Lloviu cuevavirus*, which has been genetically isolated from bats [5–7].

Prophylactic strategies include multiple immunizations to promote an effective immune response, ideally providing long-term protection by inducing long-lasting recall responses, or ring immunization, in which individuals at immediate risk of infection in an outbreak are identified and vaccinated. Ring immunization is aimed at providing short-term protection against a specific filovirus species. This strategy preferentially involves a single immunization with antigen(s) relevant to the species circulating in the outbreak, and was shown to be effective in a small scale trial during the 2013–2016 outbreak of EBOV in West Africa [8, 9]. Both strategies have merits and the use of prophylactic vaccination is predicted to have a large impact on limiting outbreaks and epidemics [10].

The filovirus envelope glycoprotein (GP) is a good vaccine antigen candidate because it is the only viral protein present on the virion surface and is used to mediate entry into the host cell. In mice, guinea pigs, and non-human primates (NHP), antibodies against GP play an important role in protection [11]. In humans, efficacy data reported from the recent ring vaccination study also points to a role of GP-specific immune responses in protection in humans [8, 9]. Research in animal models indicates that prophylactic protection against different filovirus species infections is possible by including multiple GP antigens in multivalent vaccines. Studies with monovalent and multivalent GP vaccines have shown antibody cross-reactivity in NHP [12], cross-protection of *Ebolavirus* species [13–15], and cross-protection of Marburg virus strains [13, 16–18], which may be dependent on the vaccine platform. Cross-protection capability within strains may also potentially cover as yet unknown filovirus variants.

Clinical testing of several monovalent EBOV vaccines has provided important safety, immunogenicity, and for one vaccine, efficacy information. The vesicular stomatitis virus (VSV)- based vectors vaccines are likely to be approved for use in a ring vaccination setting [8, 9, 19], but there is still a need for a prophylactic vaccine with broad specificity. Vaccines using adenovirus serotype 5 (Ad5) vectors are hampered due to pre-existing immunity to this serotype [20, 21]. However, rare seroprevalent adenoviral vector-based vaccines are capable of eliciting potent and specific humoral and cellular immune responses and are therefore promising vaccine vector candidates [22, 23]. We have developed replication-incompetent human adenovirus vectors from serotypes Ad26 and Ad35, which display a low pre-existing serological response [21]. Ad26 and Ad35 vaccine vectors induce broad, boostable humoral and cellular immune responses to encoded antigens in NHP and humans [21, 24–28]. Heterologous prime-boost regimens using different vaccine platforms, such as adenovirus in combination with modified Vaccinia virus Ankara (MVA) have shown promising results in augmenting immunogenicity [24, 29–31].

Our aim was to use stringent NHP filovirus models to test the protection and immunogenicity of a vaccine with broad specificity for both *Ebolavirus* and *Marburgvirus* filovirus species. We used a mixture of three or four adenoviral vectors, each carrying one transgene coding for the GP from EBOV Mayinga variant, SUDV Gulu variant and MARV Angola variant, with TAFV GP included in the tetravalent vaccine. These GPs were chosen to provide optimal coverage from filovirus infection, based on a phylogenetic analysis of filovirus glycoproteins [22]. It is known that there is a degree of cross-reactivity between the filovirus GPs. Although BDBV GP was not included in the multivalent vaccine, cross-reactivity as well as protection to BDBV has been observed after vaccination with EBOV GP and SUDV GP antigens [14]. The incidence of TAFV cases to date is low, and we have observed cross-reactivity to TAFV GP when using the trivalent vaccine.

We compared these multivalent vaccines in different prime-boost regimens with adenoviral vectors or in a novel multivalent combination with a MVA vaccine. We looked at GP-specific antibody and cellular responses and identified the magnitude of the immune response as a measure of protection. Our results show that multivalent vaccines given in a heterologous prime-boost immunization regimen induce immunity and provide protection, and are therefore promising candidates for a broadly protective prophylactic filovirus vaccine.

## Material and methods

### Adenoviral vaccine vector construction

Replication-incompetent, E1/E3-deleted recombinant adenoviral vectors based on adenovirus type 26 and type 35 were engineered using the AdVac® system with full-length filovirus GP. The humanized GP sequences chosen for the adenoviral transgenes stemmed from sequences of Ebola Zaire (NP_066246), Sudan Gulu (YP_138523), Tai Forest (YP_003815426) and Marburg Angola (ADM72984) strains. Rescue and manufacturing of the replication-incompetent adenoviral vectors were performed in the complementing cell line PER.C6® as described in Zahn et al, 2012 [22]. Additional Ad26 and Ad35 vectors expressing full-length EBOV GP were generated to improve expression levels by nucleotide gene optimization, that additionally contained one amino acid change V→I on position 662. These improved vectors were used in all challenge studies with MARV and EBOV. Ad5 control vector construction is described by Sullivan et al, 2006 [32].

### MVA vaccine vector construction

Primary chicken embryo fibroblast (CEF) cells used for MVA-BN-based recombinant vaccine generation and production of research grade stocks were prepared from embryonated eggs and maintained in serum free conditions.

MVA-BN-Filo (MVA-mBN226B) is a trivalent recombinant MVA (Modified Vaccinia virus Ankara, strain Bavarian Nordic [MVA-BN^®^]) based filovirus vaccine directed against Marburgvirus and Ebolavirus infection. The full-length coding sequences for GP antigens of MARV Musoke, EBOV Mayinga and SUDV Gulu as well as the nucleoprotein antigen from *Taï Forest ebolavirus* were codon optimized, synthesized (GeneArt, Regensburg, Germany) and inserted into MVA-BN. For optimal expression strong early/late promoters were chosen [33–35]. MVA-BN-Filo was generated following homologous recombination based procedures as outlined previously [36]. After insertion of the antigens into the MVA-BN genome, the deletion of the selection markers was performed by a second step of homologous recombination followed by six rounds of limiting dilution and single clone isolation to result in a genetically pure clone. This final virus clone was amplified, and a Master Virus Bank was prepared and extensively analyzed. Absence of wild type virus and of selection markers was proved by sensitive PCR and nested PCR, respectively. Sequence integrity of inserts and adjacent MVA backbone sequences were confirmed. Antigen expression was verified in transduced HeLa cells by RT-PCR and on protein level using strain specific antibodies for the three different GPs (IBT Bioservices, Gaithersburg, MD, USA) and for nucleoprotein using a polyclonal peptide antibody. Research grade product was produced in CEF and purified and concentrated in a standardized two step sucrose cushion centrifugation procedure. TCID50/ml infectious titers were determined on primary CEF cells [37].

### Ethics statement

All animal research protocols were approved by the Texas Biomedical Research Institute (TBRI) or University of Texas Medical Branch (UTMB) Institutional Animal Care and Use Committee (IACUC) in compliance with the Animal Welfare Act, Public Health Service Policy on humane care and use of laboratory animals and other federal statutes and regulations relating to animals and experiments involving animals. The studies were conducted in TBRI or UTMB’s AAALAC (International, Association for the Assessment and Accreditation of Laboratory Animal Care) accredited facility. Adult Vietnamese origin cynomolgus macaques (*Macaca fascicularis*) were at the age of 2–12 years and at a bodyweight at time of immunization of 2–12 kilograms and were obtained from Covance (Alice, Texas). All animals were males with exception of the last study where 4 females were also included. NHPs were housed singly in a 2- or 4-pack cage system. Each cage had a floor area of 0.4–0.66 m^2^ and a height of 76 cm. During the course of the study, animals were provided structural (perch), inanimate (manipulable toys) and food enrichment. Food enrichment was provided at 5–7 days per week and consisted of portions of fruits and vegetables. Euthanasia was performed in accordance with the recommended method of the Panel on Euthanasia of the American Veterinary Medical Association. Animals were sedated prior to administration of an overdose of pentobarbital sodium via the intracardiac route. Immunizations were given at the indicated doses and vaccine composition in the quadriceps femoris, or subcutaneously for MVA-BN-Filo, in a single injection with a volume of 0.5 ml. Dosing interval is indicated in the text as either 4 or 8 weeks apart. Immunizations and blood draws were performed under ketamine anesthesia.

### Filovirus challenge material and animal challenge

Viral challenges were performed with early passage challenge stocks passaged either 2 or 3 times on Vero E6 cells with Filovirus Animal Nonclinical Group (FANG) approved stocks originating from lethal human infections [38]. Challenge stocks were tested to be of identical sequence to wildtype viruses by deep sequencing and were shown to be endotoxin free. MARV Angola challenge virus was passaged 3 times (second MARV study) or 2 times (first MARV study) and was originally obtained from the 2005 outbreak [39]. SUDV Gulu was passaged 3 times on Vero cells, also shown to have a high 7U (7 polyuridine) content and derived from an outbreak in 2000 [40]. The 7U stretch is predominant in primary isolates, whereas cell culture passaging can lead to increased 8U content, which appears to reduce pathogenicity of the virus stock [41]. All EBOV studies originated from a highly lethal Kikwit-9510621 stock as shown in previous studies originating from an outbreak in 1995 [42, 43]. EBOV was passaged 3 times on Vero cells and is characterized by a low particle to pfu ratio and high 7U content, and was used in studies with 100 pfu challenge dose [42]. EBOV passaged 2 times was characterized by a low particle to pfu ratio and high 7U content, and was used in studies with 1000 pfu challenge dose [43].

NHP were located to the BSL-4 laboratory approximately three weeks post last immunization and acclimatized for 1 week. Subsequently, animals were challenged with the indicated target dose as a single intramuscular injection in 0.5 ml volume. Animals were monitored at least twice daily after challenge and more frequently when clinical signs became apparent. A clinical scoring system was used to monitor clinical signs of disease according to an IACUC approved scoring sheet. At TBRI, a score was assigned for general appearance, skin and fur, nose/mouth/eyes/head, respiration, feces and urine, food intake, petechiae, temperature and locomotor activity. These scores were recorded on a daily observation sheet and when the total value reached a critical number of 15 or more, animals were euthanized by trained and experienced personnel. At UTMB all animals were monitored daily and scored for disease progression. The scoring changes measured from baseline included posture/activity level, attitude/behavior, fruit/vegetable intake, respiration and disease manifestations such as visible rash, hemorrhage, ecchymosis. A score of greater than or equal to 9 indicated that an animal met the criteria for euthanasia (EBOV challenge study with 1000 pfu).

### Serum viral load plaque assay

Virus titration was performed by plaque assay with Vero E6 cells and serum samples. Serial dilutions of the samples were adsorbed to Vero E6 monolayers in duplicate wells. Following incubation, the cells were fixed and stained. The number of plaques in the cell monolayers was counted and the viral load was expressed as plaque forming units (pfu) per ml.

### Clinical parameters

Post-challenge time points for blood sampling and rectal temperature measurements varied by study and are listed in the supplementary table legends. The following hematology and clinical chemistry parameters are shown in the supplementary tables: granulocyte counts, levels of alanine aminotransferase, prothrombin time (PT), and activated partial thromboplastin time (aPTT). Granulocyte counts were determined in blood containing EDTA using either a VetScan HM2 Analyzer (Abaxis Inc,) or a COULTER Ac.T 5diff AL (Beckman Coulter Inc.). Clinical chemistry parameters were measured in serum using a VetScan analyzer or Piccolo Xpress (both Abaxis Inc). PT and aPTT were measured in a Coag DX analyzer (IDEXX Laboratories Inc.). Petechial rash was recorded on clinical observation sheets at least twice daily by staff blinded to study treatment.

### Filovirus glycoprotein ELISA

Filovirus-specific humoral response was determined by an enzyme-linked immunosorbent assay (ELISA), essentially as described previously [22]. MaxisorpTM 96-well plates (Nunc-Immuno) were coated over night at 4°C with Galanthus Nivalis Lectin (GNA, SIGMA Aldrich) diluted in phosphate-buffered saline (PBS, GIBCO) at a concentration of 10 μg/ml. Remaining lectin solution was removed and 200 μl PBS/10% Fetal Bovine Serum (FBS) was added for blocking at room temperature (RT) for 90 minutes. The plates were washed twice by hand with 200 μl PBS/0.2% Tween20 (Sigma-Aldrich) (PBS-T). Plates were coated with supernatant containing recombinant filovirus GP for 90 minutes at RT, and then washed by hand 3 times with 200 μl PBS-T. NHP serum was serially diluted (4-fold steps) in sample buffer starting at a dilution of 1:50 (PBS/0.2% Tween/1% FBS) in round-bottom polypropylene plates. 100 μl of diluted sample was transferred to Maxisorp 96-well ELISA plate and incubated at RT for 90 minutes. Plates were washed by hand 6 times with 200 μl PBS-T. Bound IgG was detected with goat-anti-human IgG (H+L) conjugated to HRP (Millipore USA), diluted 1:5000 in sample buffer and incubated for 1 hour at RT. Plates were washed by hand 6 times with 200 μl PBS-T. OPD (Sigma-Aldrich) was added and incubated in the dark for 10 minutes. The reaction was stopped and measured at 492 nm. IC50 values were calculated by 4-parameter curve-fit and compared against a filovirus GP strain-specific reference serum and expressed as ELISA units (EU) /ml.

### Filovirus neutralization assay

The filovirus pseudovirion neutralization assay used in this study was performed essentially as described previously [19, 44, 45] and has been shown to correlate with a wildtype neutralization assay [46]. The assay involves the use of non-replicating Vesicular stomatitis virus (VSV) ΔG-luciferase pseudovirions with surface envelopes derived from plasmids expressing the filovirus GP-of-interest. The EBOV PsVNA utilized plasmid pWRG/EBOV-Z76(opt), encoding the EBOV Mayinga GP, SUDV PsVNA utilized plasmid pWRG7077-Sudan, and MARV Angola and Musoke PsVNA utilized plasmids pWRG/MARV-ANG and pWRG7077-Musoke, respectively [47]. Pseudovirions produced using a plasmid encoding the Machupo virus envelope proteins, pWRG/MACV-GP(opt), were used as negative controls. To perform the assay, NHP sera were heat-inactivated (56°C 30 min) and diluted in media starting at 1:10 followed by 5-fold serial dilutions, and then combined with an equal volume of complete EMEM media containing 10% human complement (Sigma) and pseudovirions (10^5^ focus forming units per ml). This mixture was incubated at 4°C overnight and then inoculated (50 μl/well) onto Vero cell monolayers in clear bottom black-walled 96-well plates (Costar). Plates were then incubated for 18–24 hours and then subjected to lysis (Luciferase Kit, Promega). Luciferase reagent was added using a Tecan M200 Pro microplate reader. Raw data (relative light unit values) were exported to GraphPad Prism version 6.04 where the percentage neutralization data were normalized using cell-only and pseudovirion-only values. Percent neutralization data were fitted to a 4-parameter logistic equation using GraphPad Prism and 80% (PsVNA80) neutralization titers were interpolated from the curves for each sample. Geometric mean titers for triplicates are reported.

### Filovirus GP-reactive IFN-γ producing T cell ELISpot

Filovirus glycoprotein-specific, interferon gamma (IFN-γ)-secreting T cells were enumerated using an enzyme-linked immunospot (ELISpot) assay and isolated NHP peripheral blood mononuclear cells (PBMC). Pre-coated 96-well plates (MabTech cat# 3420M-2APT-10) with α-monkey IFN-γ capture antibody were used. Plates were washed 4 times with sterile PBS (200 μl/well) and blocked with RPMI + 10% FBS (RPMI-10) (200 μl/well) for 1 hour at 37°C and 5% CO2. PBMC were adjusted to a concentration of 2 x 10^6^ cells/ml in RPMI-10 and allowed to rest for 1 hour at 37°C and 5% CO_2_.

Filovirus GP peptide pools were used to assess T cell specificity against EBOV Mayinga, SUDV Gulu, and MARV Angola, respectively, and consist of 15-mers overlapping by 11 amino acids. GP peptide pools were divided into unique N-terminal and one C-terminal half, to limit the number of peptides per pool (43 to 58 peptides/pool at 0.4 μg/peptide/μl). Peptides that overlapped with more than nine consecutive amino acids within the EBOV Mayinga, SUDV Gulu and TAFV Ebola strains or MARV Angola and Ravn strains were combined into consensus pools (~100 peptides/pool at 0.4 microgram/peptide/microliter; EboCon, MarCon). The responses given in the figures are a combination of N- and C-terminal pools for EBOV, SUDV and TAFV. For MARV, results from N- and C-terminal pools and the MarCon pool were combined.

Peptide pool working solutions were prepared in RPMI-10 at a final concentration of 2 μg/ml and 50 μl/well. The negative control contained 1.5% DMSO in RPMI-10 and the positive control 1 μg/ml α-CD3 antibody in RPMI-10. All samples were run either in duplicates or triplicates. The block-buffer was removed from plates and peptide pools, or controls (50 μl/well) were added to the plates, followed by 100 μl/well of cell suspension (2 x 10^5^ cells). Plates were covered with a sterile lid and wrapped in aluminum foil and incubated for 20 ± 1 hours at 37°C and 5% CO_2_. The cell suspension was removed and wells were washed 5 times with PBS (200 μl/well) at RT. Released IFN-γ was detected by adding 100 μl/well alkaline phosphatase conjugated IFN-γ detector antibody (1:200 in PBS + 0.5% FBS). Plates were sealed and incubated for 2 hours at RT. Plates were washed 5 times with PBS (200 μl/well). NBT/BCIP-plus was filtered through a 0.45 um filter and 100 μl of the solution was added to each well. Spots were developed for 15 minutes in the dark at RT. The development was stopped by washing extensively with tap water. Plates were air dried and spots were counted on an AELVIS ELISpot reader.

### Statistical analysis

Paired t-tests compared pre- and post-boost antibody titers. Statistical analysis was performed using R software.

## Results

### Immunogenicity of a tetravalent GP vaccine

**Fig 1:**
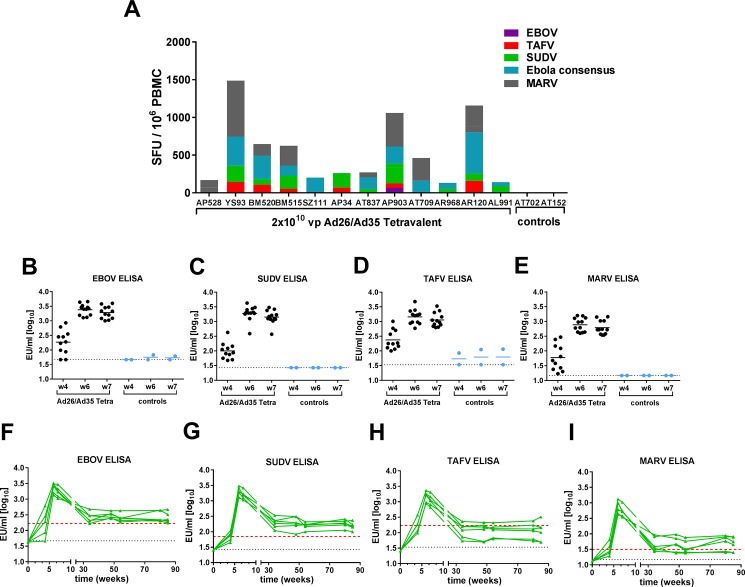
Immunity to filovirus glycoprotein using a tetravalent vaccine in a heterologous (Ad26-Ad35) regimen. Cynomolgus macaques were immunized with heterologous Ad26-prime at week 0 and Ad35-boost at week 4 with a tetravalent vaccine (total dose 8x10^10^ vp) or empty Ad vectors as controls. (A) Cellular immune response 2 weeks after boost immunization using IFNγ ELISpot after stimulation with the indicated filovirus GP peptide pools. (B-E) Humoral immune responses after prime and boost immunizations as measured by ELISA for the indicated filovirus GP. Bars designate the mean response and each circle represents an individual animal. The black dotted line represents the lower limit of detection. (F-I) Humoral immune responses over 85 weeks measured by ELISA for the indicated filovirus GP. Each green line represents an individual animal, the red dotted line is the average response after priming, the black dotted line represents the lower limit of detection.

We first asked if a tetravalent adenoviral vector-based filovirus GP vaccine could elicit specific immune responses against GP from the different filovirus species in NHP. Previous studies demonstrated the immunogenicity of the individual constructs in mice [22]. Our studies were performed with cynomolgus macaques because this NHP model is considered the gold standard for testing filovirus vaccine candidates. We used a heterologous prime-boost strategy with Ad26 and Ad35 vectors. S1 Table shows the study designs and vaccine and filovirus challenge doses. Vectors carrying transgenes for MARV Angola GP, EBOV Mayinga GP, SUDV Gulu GP, or TAFV GP were mixed together using 2x10^10^ viral particles (vp) of each vector to create a tetravalent vaccine. Animals were primed with the Ad26 vectors and boosted 4 weeks later with the Ad35 vectors. Empty adenovirus vectors with no transgene were used as negative controls. An ELISpot assay was used to determine filovirus GP-specific IFNγ+ T cell responses from PBMC taken 2 weeks after the Ad35 boost. PBMC were incubated with peptide pools derived from EBOV Mayinga GP, SUDV Gulu GP, TAFV GP and MARV Angola GP. The number of IFNγ+ spot forming units (SFU) in response to *Ebolavirus* GP strain-specific 15mer peptide pools, to an *Ebolavirus* GP consensus peptide pool, and the MARV peptide pool, are shown for each animal in Fig 1A. In each animal, the tetravalent vaccine elicited a specific T cell response against at least one filovirus GP. Recognition of both *Ebolavirus* and *Marburgvirus* GP peptides was seen in 7 out of 12 animals. The magnitude of the response varied per animal, with a total response against the combination of all 4 GP antigens of between 133 and 1488 IFNγ+ SFUs/10^6^ PBMC. There was no GP-specific response in control animals vaccinated with empty vectors.

The antibody response to filovirus GP was measured in serum taken 4 weeks after priming with Ad26, and 2 weeks and 3 weeks after the Ad35 boost, as well as during 85 weeks post prime in a subset of NHP. Antibodies to EBOV GP, SUDV GP, TAFV GP and MARV GP were measured by ELISA. Priming with the tetravalent Ad26 vaccine elicited a specific antibody response in most animals (Fig 1B–1E, week 4). A boost with the tetravalent Ad35 vaccine led to an increase in theantibody titers, ranging from 19-fold increase for SUDV GP to a 6-fold increase for TAFV GP (Fig 1B–1E, week 6). The antibody response to EBOV GP, SUDV GP and MARV GP was sustained at levels above the mean priming response over 85 weeks studied (Fig 1F, 1G and 1I). The long-term antibody response to TAFV GP was generally below the mean titer measured after priming, but titers remained detectable in each animal (Fig 1H). This indicates that fewer long-lived plasma cells were generated at the time of boost vaccination.

The tetravalent vaccine in a heterologous Ad26-Ad35 prime-boost regimen elicited filovirus GP-specific T cell and antibody responses. Due to the multivalent nature of the vaccine, the immune response was directed to each species-specific GP, indicating this multivalent vaccine has the potential to provide protection against different filovirus species. We tested this multivalent vaccine in three different filovirus challenge models, using otherwise lethal infections of NHP with MARV, SUDV and EBOV, to see if the vaccine could provide broad protection.

### Tetravalent vaccine in immunogenicity and protection to Marburg virus

We tested the ability of the tetravalent vaccine to provide protection in cynomolgus macaques receiving a lethal filovirus challenge with 1000 pfu of MARV Angola. While a mixture of 2x10^10^ vp of each Ad-GP was sufficient to elicit specific antibody and T cell responses (Fig 1) we did not know if this response was protective. Therefore, we performed lethal challenge studies after vaccinating macaques with either 2x10^10^ vp (low dose, total dose 8x10^10^ vp) or 1x10^11^ vp (high dose, total dose 4x10^11^ vp) of each Ad-GP in a tetravalent vaccine. Animals were primed with Ad26-GP vectors and boosted with Ad35-GP vectors 4 weeks later. In addition, Ad26-/Ad35-MARV GP was used as a monovalent vector at the 1x10^11^ vp high dose, and one group received a high dose of empty Ad26-Ad35 vectors as a negative control. The MARV challenge was performed 4 weeks after the boost vaccination.

**Fig 2:**
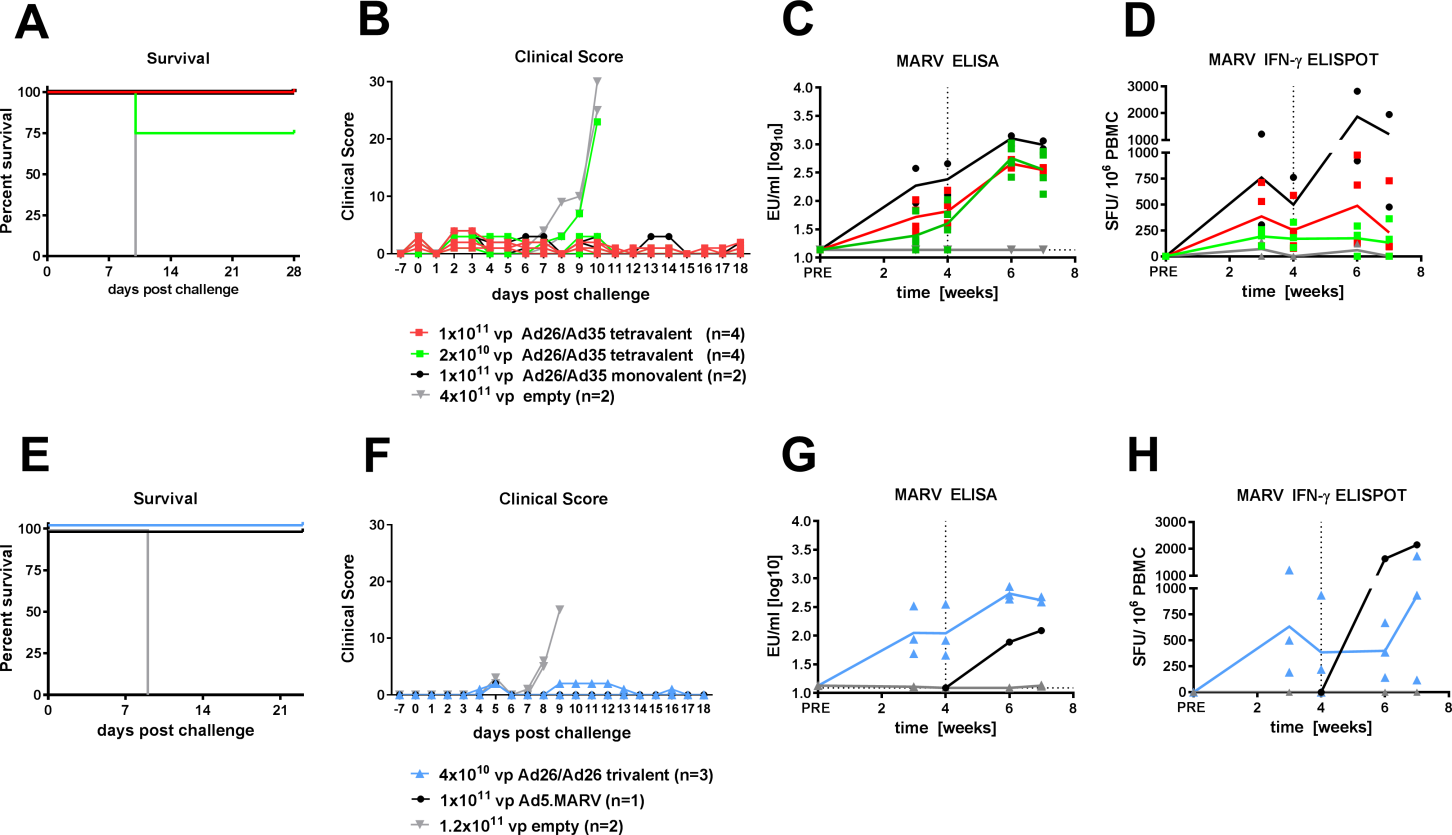
Immunogenicity of heterologous (Ad26-Ad35) tetravalent and homologous (Ad26) trivalent vaccine regimens and protection from MARV Angola challenge. (A-D) Cynomolgus macaques were immunized with heterologous Ad26 prime at week 0 and Ad35 boost at week 4 with a tetravalent vaccine, or a monovalent Ad26 MARV GP vaccine, or empty Ad vectors, at the doses indicated. (E-H) Cynomolgus macaques were immunized with homologous trivalent Ad26 prime at week 0 and Ad26 boost at week 4, or a monovalent Ad5 MARV GP vaccine (prime only at week 4), or empty Ad vectors, at the doses indicated. A challenge with 1000 pfu MARV Angola was given at week 8. (A+E) Kaplan-Meier representation of survival. (B+F) Clinical scoring of individual animals after lethal challenge. (C+G) Humoral immune response over time measured by MARV GP-specific ELISA. Horizontal dotted line represents the lower limit of detection. Solid lines indicate the group mean. (D+H) Cellular immune response to MARV GP peptide pool by IFNγ ELISpot. Vertical dotted lines indicate the time of boost immunization. Solid lines indicate the group mean.

Cynomolgus macaques challenged with a lethal dose of MARV were fully protected by prophylactic vaccination with the high dose Ad26-Ad35 multivalent vaccine (4/4 animals) and monovalent vaccine (2/2 animals) (Fig 2A). Vaccination with the lower dose provided partial protection from lethal challenge (3/4 animals). Vaccination with empty Ad26/Ad35 vectors did not provide protection (0/2 animals) and time to death was similar to that of control animals from previous studies receiving MARV challenge. The clinical scoring of disease symptoms remained low in protected animals (Fig 2B) and the animals that did not survive consistently showed symptoms of hemorrhagic fever with changes in body temperature, elevated alanine aminotransferase, granulocytosis, changes in coagulation profile, petechial rash, and viremia (S2 Table).

We further investigated the immune response to MARV GP in these animals. Anti-MARV GP titers were measured in the serum of animals 3 and 4 weeks after priming with Ad26, and 2 and 3 weeks after the Ad35 boost (Fig 2C). After priming, at week 4, the mean serum titer of anti-MARV GP antibody was highest in the 2 animals receiving the monovalent vaccine, and lowest in the low dose tetravalent vaccine group, where the one seronegative animal succumbed to lethal challenge. The anti-MARV GP antibody titers significantly increased after boosting in both tetravalent groups (Week 4 versus Week 6 paired T test: high dose tetravalent p = 0.0096; low dose tetravalent p = 0.001). The mean antibody titers in the low and high dose tetravalent groups were similar at 3 weeks after boost vaccination (week 7), although the range in antibody titers was larger in the low dose vaccine group. The mean antibody titer in the 2 animals receiving the monovalent vaccine remained higher than titers from animals receiving the multivalent vaccine.

We also investigated MARV GP-specific T cell responses using IFNγ ELISpot (Fig 2D). There was a large variation in the number of SFUs at each time point, especially in the high dose tetravalent vaccine and monovalent vaccine treated groups. Given the outbred nature of this animal model, such variation in cellular responses is expected. In the high dose tetravalent vaccine group there was a trend to a higher number of IFNγ-producing cells after boosting at week 4, but this effect was diminished at week 7; the boosting effect was more pronounced in the monovalent vaccine group. In the low dose tetravalent group the mean SFU count remained low and did not change over time. Although a MARV challenge was performed in this study, T cell and humoral responses to all the GP antigens in the tetravalent vaccine were measured (S1 Fig). Similar to the response to MARV GP, there was a specific and boostable antibody response to GP from EBOV, SUDV and TAFV. All NHP receiving the tetravalent vaccine showed IFNγ responses to both *Ebolavirus* and *Marburgvirus* GP peptides.

### Trivalent vaccine in immunogenicity and protection to Marburg virus

We concluded that the immune response induced by the tetravalent vaccine at both doses tested was sufficient to provide protection against lethal challenge, with complete protection at the higher dose and partial protection at the lower dose. We also tested a trivalent vaccine in the MARV challenge model. The trivalent vaccine contained 4x10^10^ vp each of Ad26 carrying the EBOV GP, SUDV GP, or MARV GP transgenes (total dose 1.2x10^11^ vp). The TAFV GP vector was excluded due to the extremely low number of humans infected and cross-reactivity of EBOV GP and SUDV GP to TAFV GP (S4 Fig). We included MARV GP carried in an Ad5 vector at 1x10^11^ vp as a control. In addition to a reduced valency vaccine, we simplified the regimen further by using a homologous Ad26 prime and Ad26 boost. This trivalent vaccine regimen protected against lethal challenge with MARV Angola (3/3), as did the single vaccination with Ad5 MARV GP monovalent vaccine (1/1) given 4 weeks before challenge (Fig 2E). The clinical scores for disease symptoms remained low in the protected animals (Fig 2F, and S3 Table for a display of clinical parameters for each NHP). MARV GP-specific antibodies increased in 2 of 3 animals after boost immunization (Fig 2G) and the humoral immune response to the trivalent vaccine was similar in magnitude to the Ad26-Ad35 vector heterologous prime-boost tetravalent vaccine response (Fig 2C). The MARV GP-specific IFNγ+ T cell responses showed a large variation in the number of SFUs at each time point, and the mean SFU count generally remained relatively constant over time after prime immunization (Fig 2H).

### Trivalent vaccine in immunogenicity and protection to Sudan virus

The tetravalent and trivalent vaccines could protect NHP against lethal infection with Marburg virus. We then tested our trivalent vaccine for protection from a lethal challenge with *Ebolavirus* species, which are generally considered more stringent challenge models [48], because disease progression is faster, and *Ebolavirus* species cause greater morbidity than challenge with *Marburgvirus*.

**Fig 3:**
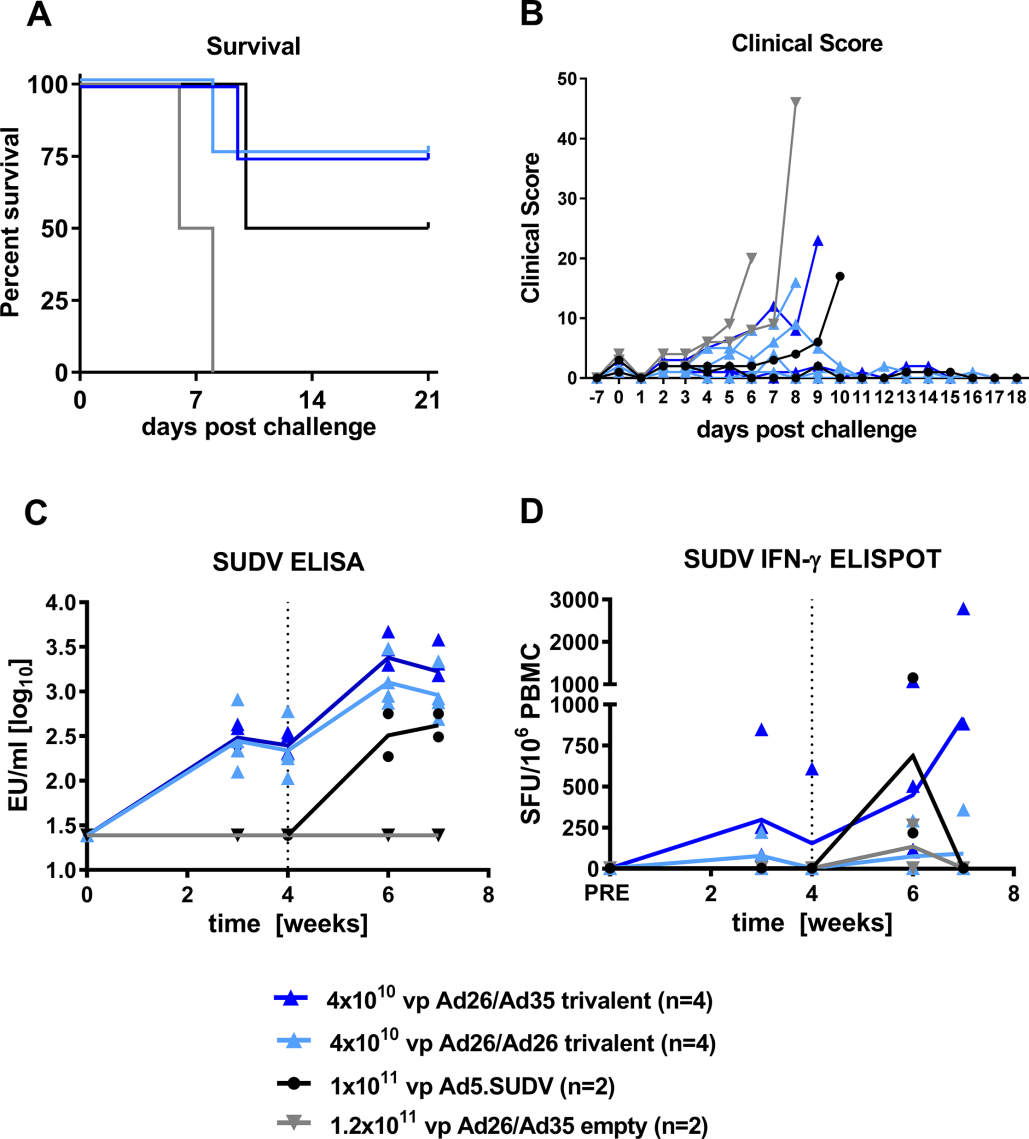
Immunogenicity of heterologous (Ad26-Ad35) and homologous (Ad26-Ad26) trivalent vaccine regimen and protection from SUDV Gulu challenge. Cynomolgus macaques were immunized with heterologous (Ad26-Ad35) or homologous (Ad26-Ad26) prime at week 0 and boost at week 4 with a trivalent vaccine, or a monovalent Ad5 SUDV GP vaccine (prime only at week 4), or Ad empty vectors at the doses indicated. A challenge with 1000 pfu SUDV Gulu was given at week 8. (A) Kaplan-Meier representation of survival. (B) Clinical scoring of individual animals after lethal challenge. (C) Humoral immune response over time measured by SUDV GP-specific ELISA. Solid lines indicate the group mean. (D) Cellular immune response to SUDV GP peptide pool measured by IFNγ ELISpot. Vertical dotted lines indicate the time of boost immunization. Solid lines indicate the group mean.

We vaccinated cynomolgus macaques using the 4 x 10^10^ vp trivalent vaccine (total dose 1.2x10^11^ vp) or 1.2 x 10^11^ vp empty vectors, with a heterologous Ad26-Ad35 or homologous Ad26-Ad26 vector prime-boost schedule. A control group was primed at week 4 with 1x10^11^ vp Ad5 carrying the SUDV GP transgene. The animals were given a lethal challenge with 1000 pfu SUDV Gulu. The 2 NHP receiving empty Ad vectors both succumbed to infection showing signs of hemorrhagic fever and viremia (S4 Table). Heterologous and homologous prime-boost vaccination with the trivalent vaccine provided protection in 3/4 animals each (Fig 3A). One surviving animal in the homologous priming group had clinical signs of disease on day 8 post challenge but recovered (Fig 3B). Surprisingly, one of the two positive control animals also succumbed to infection, which is inconsistent with reported data [17] indicating that the challenge model chosen here was highly stringent. Priming with trivalent Ad26 filovirus GP vectors induced an antibody response to SUDV GP that significantly increased after the boost immunization (Fig 3C; Week 4 versus Week 6 paired T test: Ad26-Ad26 p = 0.02; Ad26-Ad35 p = 0.005). Heterologous prime-boost vaccination with the trivalent vaccine appeared to be better than the homologous prime-boost vaccination at inducing an IFNγ+ T cell response to SUDV GP peptides, although there was a large range in SFU counts (Fig 3D) given the outbred nature of these animals. A comparison of the antibody responses to SUDV GP, EBOV GP and MARV GP indicated that heterologous prime-boost may be superior to homologous prime-boost in inducing GP-specific antibodies (Fig 3C and S2B Fig), and in inducing higher numbers of IFNγ-producing cells (Fig 3D and S2A Fig), although no formal comparisons were made due to limited group sizes.

### Trivalent and tetravalent vaccine in immunogenicity and protection to Ebola virus

**Fig 4:**
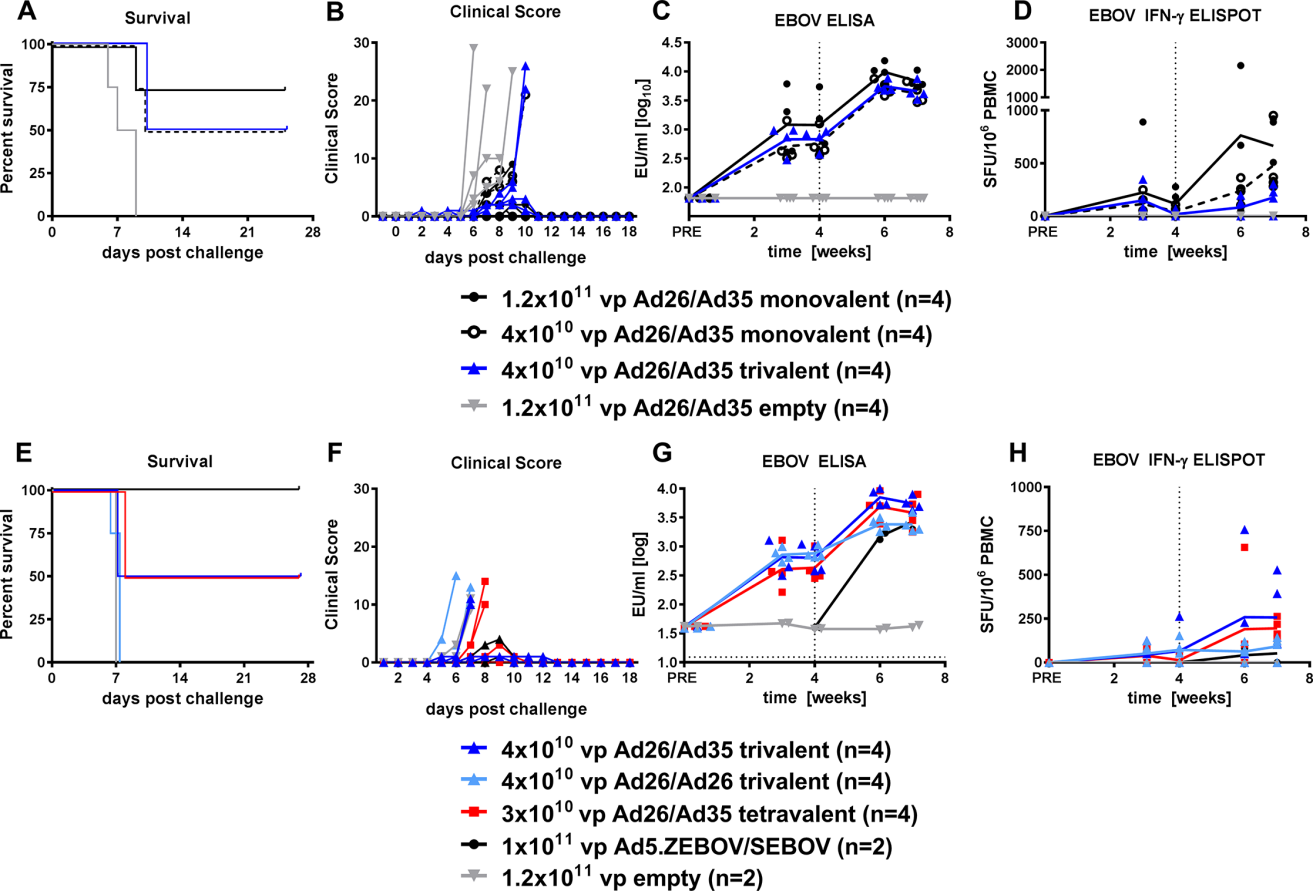
Immunogenicity of tetravalent, trivalent, and monovalent vaccines and protection from EBOV Kikwit challenge. (A-D) Cynomolgus macaques were immunized with heterologous Ad26-prime at week 0 and Ad35 boost at week 4 with a trivalent vaccine, or a monovalent vaccine (Ad.ZEBOV), or empty Ad vectors, at the doses indicated. A challenge with 100 pfu EBOV Kikwit was given at week 8. (E-H) Cynomolgus macaques were immunized with a heterologous (Ad26-Ad35) or homologous (Ad26-Ad26) prime at week 0 and boost at week 4 with a trivalent vaccine, or tetravalent vaccine, or empty Ad vectors, or a bivalent Ad5 EBOV GP + SUDV GP vaccine (prime only at week 4), at the doses indicated. A lethal challenge with 1000 pfu EBOV Kikwit was given at week 8. (A+E) Kaplan-Meier representation of survival. (B+F) Clinical scoring of individual animals after lethal challenge. Clinical score criteria for euthanasia was 15 in (B) and 9 in (F). (C+G) Humoral immune response over time measured by EBOV GP-specific ELISA. Horizontal dotted line represents the lower limit of detection. Solid lines indicate the group mean (D+H) Cellular immune response to EBOV GP peptide pool by IFNγ ELISpot. Vertical dotted lines indicate the time of boost immunization. Solid lines indicate the group mean.

The trivalent vaccine was then tested for the ability to protect against lethal infection with 100 pfu EBOV Kikwit. Cynomolgus macaques were vaccinated with 4 x 10^10^ vp trivalent vaccine (total dose 1.2x10^11^ vp) using an Ad26-Ad35 heterologous prime-boost schedule with a 4 week spacing. A heterologous prime-boost was also performed with both low dose (4x10^10^ vp) or high dose (1.2x10^11^ vp) of a monovalent vector expressing EBOV GP (Ad-ZEBOV) to compare the immune responses of monovalent and multivalent vaccines. Vaccination with the trivalent vaccine provided protection in half (2/4 animals) of the vaccinated animals (Fig 4A). The high dose and low dose monovalent heterologous prime-boost vaccines with EBOV GP gave protection in 3/4 and 2/4 animals respectively (Fig 4A). An analysis of the antibody response showed that the trivalent vaccine (containing 4x10^10^ vp Ad-ZEBOV) and the monovalent Ad-ZEBOV (4x10^10^ vp) induced similar titers of EBOV GP specific antibodies, indicating there was no immune interference from the other vectors in the trivalent vaccine (Fig 4C). Titers in all groups were significantly higher after booster immunization (Week 4 versus Week 6 paired T test: Ad26-Ad35 trivalent p = 0.0030; Ad26-Ad35 monovalent low dose p = 0.0028; Ad26-Ad35 monovalent high dose p = 0.0311). The mean antibody titer appeared to be slightly higher after immunization with the high dose 1x10^11^ vp monovalent Ad-ZEBOV (Fig 4C). The trivalent vaccine also induced antibodies to SUDV and MARV GP (S3B Fig). The counts for EBOV GP peptide-specific IFNγ+ T cells were variable and generally low (Fig 4D) with exception of the high dose monovalent vaccine group. Further analysis of the cellular response showed that Ad-ZEBOV induced EBOV GP-specific responses, while the trivalent vaccine induced cellular responses to EBOV, SUDV and MARV GP peptides (S3A Fig).

The trivalent vaccine regimen was repeated and directly compared with the tetravalent vaccine using a dose of 1000 pfu EBOV Kikwit. A control group received a prime only vaccine at week 4 with Ad5 vectors carrying the EBOV GP and SUDV GP transgenes, because priming with this vector was shown to give complete protection after EBOV challenge [26]. Complete protection was confirmed in this study (Fig 4E), although in contrast to previously reported studies, one animal did show disease symptoms (Fig 4F). Vaccination with the trivalent or tetravalent vaccine (both 1.2x10^11^ vp total dose) given in a heterologous Ad26-Ad35 prime-boost regimen provided protection in half of the vaccinated animals (2/4 per group), thereby reproducing the findings in the previous EBOV challenge study with the trivalent vaccine, but using a higher challenge dose. In contrast, there was no protection in the animals receiving the trivalent vaccine in the homologous Ad26-Ad26 regimen (0/4, Fig 4E). There was a significant increase in EBOV GP antibody titers after boost vaccination (Week 4 versus Week 6 paired T test: Ad26-Ad26 trivalent p = 0.0011; Ad26-Ad35 trivalent p = 0.0004; Ad26-Ad35 tetravalent p = 0.0076). The EBOV GP specific antibody response was similar between the groups receiving the trivalent and tetravalent vaccines given in the heterologous regimen (Fig 4G), as were the antibody responses to SUDV GP and MARV GP (S4B Fig). The trivalent vaccines were able to stimulate a cross-reactive antibody response to TAFV GP after boost immunization, with higher cross-reactivity induced after heterologous boost (S4B Fig). The counts for EBOV GP peptide-specific IFNγ+ T cells were variable and generally low (Fig 4H). The individual cellular responses to EBOV, SUDV, TAFV and MARV GP peptides were variable (S4A Fig). Ad26-Ad35 heterologous regimens induced higher immune responses compared to the homologous vaccination regimens.

A summary of the clinical parameters from the EBOV challenge studies is shown in S5, S6 and S7 Tables, showing serum viral load, petechial rash, rectal temperature, ALT, granulocyte counts, PT and aPTT. Serum viral load and petechia at the analyzed time points were only present in NHP that succumbed to infection. Non-survivors had evidence of viral load and changes in multiple clinical parameters consistent with filovirus disease; all except one animal displayed petechiae.

### Combination of trivalent Ad26 and MVA-BN-Filo in protection and immunogenicity to Ebola virus

Finally, we used the EBOV lethal challenge model in a pilot study with two animals per group to compare the trivalent Ad26-Ad35 heterologous vaccine regimen with a prime-boost heterologous regimen using trivalent Ad26 with the MVA vectored MVA-BN-Filo. MVA-BN-Filo is a multivalent vaccine expressing the GP of the EBOV Mayinga variant, the GP of the SUDV Gulu variant, the GP of the MARV Musoke variant (93% amino acid homology with MARV Angola GP), and the TAFV nucleoprotein. This vaccine is currently undergoing clinical trials in combination with monovalent Ad26.ZEBOV [24, 31]. The boost vaccination was given 8 weeks after priming because a longer interval between doses of Ad and MVA has been shown to elicit a more potent immunological response [49]. The Ad26/Ad35 monovalent group received a boost at 4 weeks after priming. Monovalent and trivalent Ad immunizations were given at a total dose of 1.2x10^11^ vp. MVA-BN-Filo was given subcutaneously at 5x10^8^ of the 50% tissue culture infective dose (TCID50).

**Fig 5:**
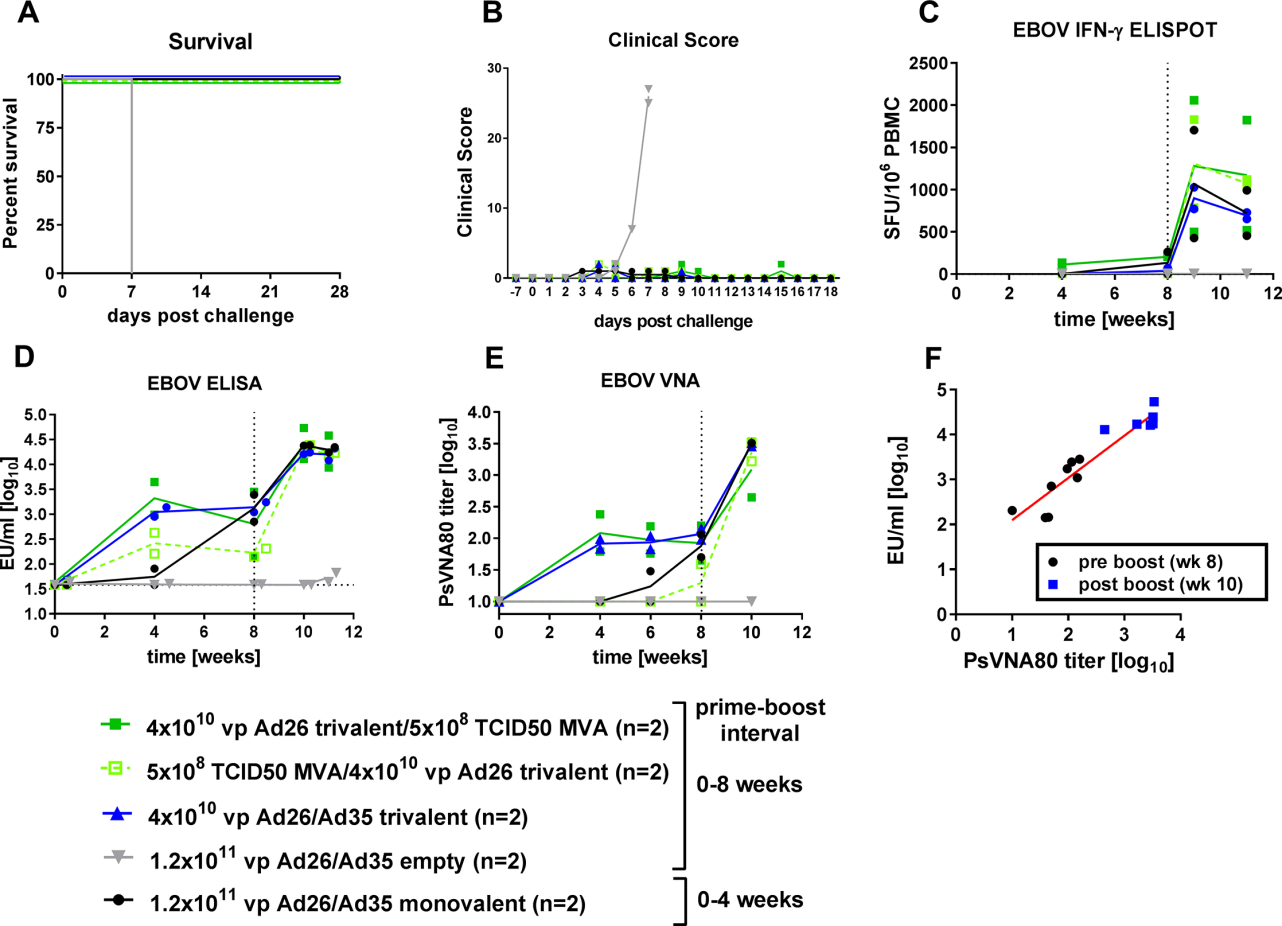
Immunogenicity of heterologous (Ad26-Ad35, Ad26-MVA-BN-Filo or MVA-BN-Filo-Ad26) trivalent, and Ad26-Ad35 monovalent vaccines and protection from EBOV Kikwit challenge. Heterologous (Ad26-Ad35, Ad26-MVA or MVA-Ad26) prime at week 0 and boost at week 8 with a trivalent vaccine, or empty Ad26-Ad35 vectors, at the doses indicated. One group received a heterologous Ad26-Ad35 prime at week 4 and boost at week 8 of monovalent vaccine (Ad.ZEBOV). A challenge with 100 pfu EBOV Kikwit was given at week 12. (A) Kaplan-Meier representation of survival. (B) Clinical scoring of individual animals after lethal challenge. (C) Cellular immune response to EBOV GP peptide pool by IFNγ ELISpot. (D) Humoral immune response over time measured by EBOV GP-specific ELISA. Horizontal dotted line represents the lower limit of detection. (E) Neutralizing antibody response over time measured by pseudovirion neutralization assay. (C-E) Solid lines indicate the group mean. (F) Correlation of pre-boost (black symbols) and post-boost (blue symbols) ELISA titers with virus neutralization antibody titers. Vertical dotted lines indicate the time of boost immunization.

A lethal challenge of 100 pfu EBOV Kikwit was given 4 weeks after the boost vaccination. All heterologous combinations (Ad26-Ad35, MVA-Ad26, Ad26-MVA) with a trivalent vaccine approach gave complete protection to a lethal challenge with 100 pfu EBOV Kikwit (Fig 5A and 5B). In contrast to our previous experiments, EBOV GP peptide-specific IFNγ+ T cell responses, measured from week 4, were markedly increased after boosting in all GP vaccine treatment groups (Fig 5C). We looked at EBOV GP-specific antibody titers and at the titer of virus neutralizing antibodies to EBOV GP. In all groups, EBOV GP-specific antibody responses were higher after boosting than after priming. The mean antibody titer at 2 and 3 weeks after boost vaccination was similar across all treatment groups receiving the GP transgene (Fig 5D week 10 and 11). The presence of virus neutralizing antibody closely mirrored the development of anti-EBOV GP antibodies (Fig 5E). There was also a strong neutralizing antibody response to SUDV, and neutralizing antibodies were induced after boost vaccination for MARV in the groups receiving a multivalent Ad26 prime (S5B Fig). When all groups were combined, there was a linear correlation between anti-EBOV GP total antibodies and EBOV GP neutralizing antibodies at week 8 (pre-boost) and week 10 (2 weeks post-boost) time points after vaccination with the GP transgene (Fig 5F).

### Magnitude of the GP-specific immune response as a predictor of protection

**Fig 6:**
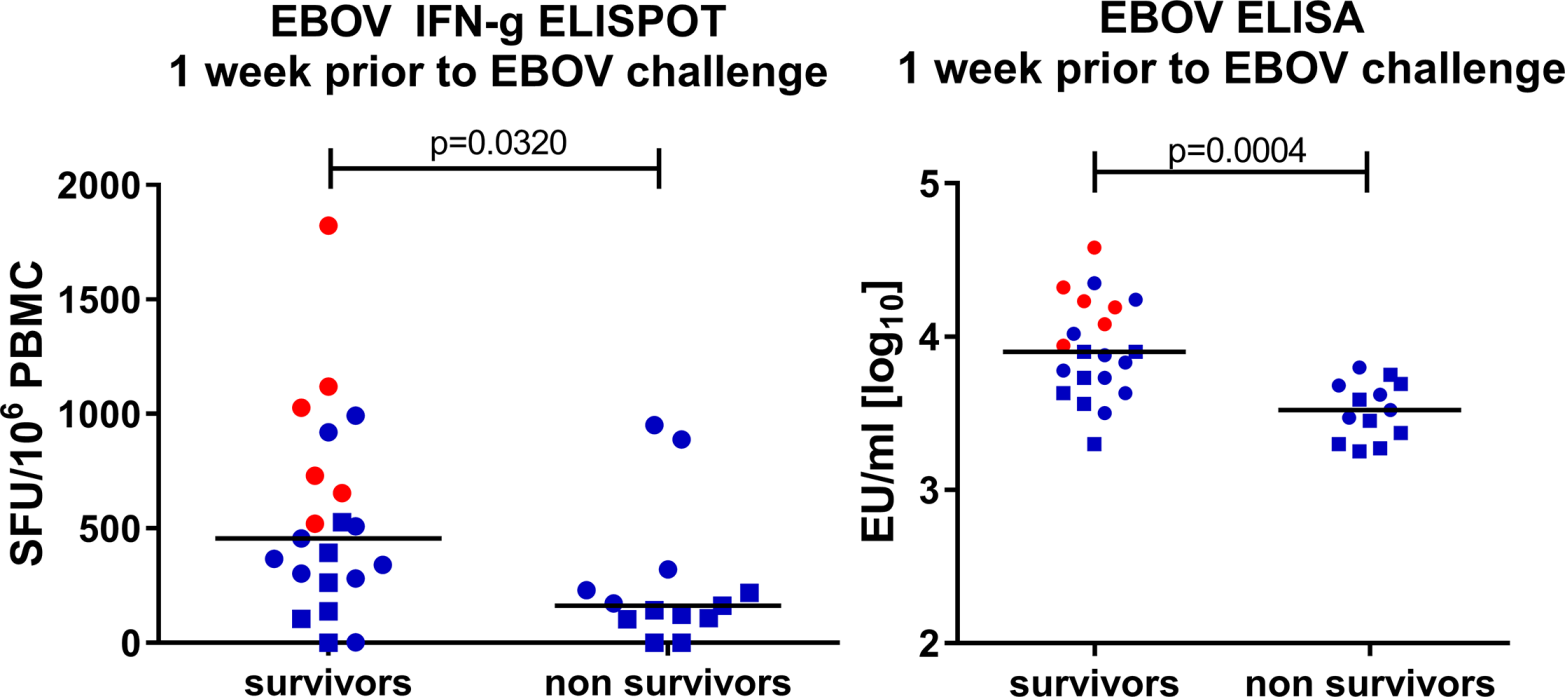
Comparison of EBOV GP-specific cellular and antibody responses 1 week prior to EBOV challenge in challenge survivors and non-survivors. Summary of data from lethal challenge experiments with EBOV Kikwit shown in Fig 4 and Fig 5. Animals receiving vectors with EBOV GP in mono- or multivalent regimens are shown. Dots: 100 pfu challenge, squares: 1000 pfu challenge; blue: 0–4 week regimen, red: 0–8 week regimen. (A) Cellular immune response to EBOV GP peptides measured by IFNγ ELISpot. (B) Humoral immune response measured by EBOV GP-specific ELISA. p-values were calculated with the exact Wilcoxon rank sum test, and adjusted for multiplicity for each of the 2 pairs of tests (ELISpot and ELISA) using the Bonferroni adjustment.

Combining the data from the EBOV Kikwit lethal challenge studies of adenoviral vector or/and MVA immunized NHP revealed that EBOV GP-specific humoral responses were significantly higher (p = 0.0004, and p = 0.0026 when MVA-boosted animals were excluded) in survivors than non-survivors at the last time point measured prior to challenge (Fig 6B). Cellular responses measured by ELISpot were also higher in survivors than non-survivors (p = 0.0320, Fig 6A; and p = 0.1428 when MVA-boosted animals were excluded).

## Discussion

This is the first report on NHP efficacy studies evaluating a multivalent filovirus vaccine candidate based on human Ad26, Ad35 and MVA vectors. All regimens tested induced a strong and durable humoral immune response against all vaccine antigens, and a cellular response against all antigens. A heterologous Ad26-Ad35 prime-boost vaccination regimen could give full protection against MARV and EBOV challenge and partial protection against SUDV challenge in stringent NHP challenge models. Full protection against EBOV challenge was also achieved when Ad26 and MVA were combined in a prime-boost vaccination regimen. Such an Ad26/MVA regimen with monovalent Ad26 priming is currently being explored in clinical trials as a potential vaccine candidate against EBOV infection.

Recent reports have indicated that compared to human infection and case fatality rates, NHP filovirus challenge model stringency is high, and dependent on the challenge inoculum used [42, 50]. The Filovirus Animal Non-clinical Group (FANG) has made recommendations to standardize the animal model and challenge material used for evaluation of vaccines [38]. Here we used recommended low passage, stringent and well characterized EBOV challenge inoculum with a high degree of 7 uridines at a critical transcription site important for virulence. We used two different challenge doses of EBOV, 100 pfu and 1000 pfu, and found no difference in the time to death in control animals receiving empty vectors, and also no obvious differences in clinical parameters of non-survivors (S5, S6 and S7 Tables). Indeed, immunization with Ad5.ZEBOV/SEBOV is known to protect from death and morbidity against challenge with 1000 pfu EBOV even with 10-fold lower vaccine dose [26, 51], yet one Ad5-vaccinated NHP showed signs of clinical symptoms after 1000 pfu EBOV in our studies, showing the stringency of the EBOV challenge virus model used in the studies presented here. The number of surviving NHP given a prime-boost regimen with trivalent Ad26/Ad35 was the same in 100 pfu and 1000 pfu EBOV challenge studies. This is consistent with a report where titration of high particle/pfu challenge stocks showed little difference in clinical scores and survival rates over a 100-fold dose range [42]. Furthermore, a dose effect using monovalent Ad26 vectors was observed after 100 pfu EBOV challenge. Therefore, the 100 pfu EBOV NHP challenge dose appears to be sufficiently stringent to test vaccine efficacy. It is of note that such stringent NHP challenge models may underestimate the efficacy of vaccines in the human population.

The use of the multivalent vaccine did not show overt immune interference between the included strains in these studies. Although there was a trend towards higher MARV GP-specific immune responses after monovalent vaccination (Fig 2C and 2D), monovalent and trivalent vaccines giving Ad-ZEBOV at the same dose elicited similar EBOV-specific humoral and cellular responses (Figs 4C and 4D and 5C–5E). Similarly, the trivalent and tetravalent vaccines given at the same total dose showed comparable humoral and cellular responses, and the trivalent vaccine also displayed cross-reactivity to TAFV GP. Although not tested here, cross-reactivity as well as protection to BDBV has also been observed after expression of EBOV GP and SUDV GP antigens [14]. Since filovirus GP drift is limited, with little or no evolution in humans [52, 53], the likelihood that a trivalent vaccine will protect against future outbreaks is high. In addition, we favor a heterologous prime-boost regimen because heterologous Ad26-Ad35 prime-boost appeared to be superior to homologous Ad26-Ad26 prime-boost in terms of immunogenicity, and for EBOV infection, a higher degree of protection. Interestingly in humans a homologous Ad26 prime-boost regimen with an HIV Env glycoprotein antigen was shown to induce a high degree of humoral and cellular responses [28]. The multivalent heterologous Ad26-MVA-BN-Filo combination was also immunogenic. The induction of virus neutralizing antibodies against all GPs contained in the vaccine shows the potential of this multivalent approach.

Recent published results in humans have confirmed the immunogenicity and safety of the Ad26.ZEBOV and MVA-BN-Filo combination [24, 31]. In addition, we have shown that the heterologous (Ad26-Ad35) prime-boost strategy has the potential to induce long-lasting immunity in NHP to all antigens in the tetravalent vaccine (Fig 1F–1I). Durable antibody responses were also seen in a phase I clinical trial using the heterologous Ad26.ZEBOV and MVA-BN-Filo prime-boost regimen when measured up to one year post-prime immunization [24, 31].

The translation of a vaccine-induced immune response in NHP to the induced response in humans will be challenging as it has been shown for other filovirus vaccines that the immune response in humans was lower than in NHP [29]. In the case of the filovirus vaccines, this is of high significance as efficacy studies in humans will likely not be feasible due to the sporadic nature of outbreaks. Efficacy in humans might not be demonstrable until after licensure, requiring that vaccine registration is supported by NHP efficacy studies under the Animal Rule, for which a translation of the NHP vaccine-induced immune response to humans is essential. Data generated in ongoing human clinical studies with a monovalent Ad26/MVA based vaccine, as well as future multivalent studies will allow evaluation of the full value of the NHP model.

Universal immunological correlates of vaccine-mediated protection from filovirus disease have not been defined. Both humoral and cellular responses are considered necessary for protection, although the relative contribution of each is not fully understood [54, 55]. Immunization with a trivalent mixture of vectors carrying transgenes coding for EBOV, SUDV or MARV GP induced robust and specific humoral immune responses, and the responses were potentiated after boost immunization. Moreover, our results showed that the humoral immune response measured by ELISA correlated with VNA for EBOV (Fig 5F), and therefore the antibody response is potentially honed to control infection through virus neutralization. We also saw that antibody titers prior to challenge could be used to distinguish protective versus non-protective immunization in our NHP EBOV model, with higher antibody titers associated with protection. Furthermore, post-exposure treatment with antibodies protected against lethal EBOV and MARV infections in rhesus macaque models, indicating that antibodies are sufficient to protect against lethal disease [56–58]. Cellular responses to the filovirus GPs were also generated and a higher frequency of IFNγ-producing cells was associated with survival in EBOV challenged animals. For MARV and SUDV the numbers of animals in the studies did not allow for exploration of the relative contribution of humoral or cellular immune responses to protection. However, there appears to be a stronger role for antibody responses as a correlate of protection against filovirus [59–61].

Our results demonstrate that the development of a multivalent filovirus vaccine with cross-species protection is feasible. Adenoviral and MVA-based vaccines can already be manufactured on a large scale. One of our vaccine candidate regimens demonstrated long-term immunogenicity in NHP and also in humans using an Ad26.ZEBOV/MVA-BN-Filo vector combination [24, 31]. A multivalent filovirus vaccine would be optimal for prophylactic administration, for example, of populations who are deemed to be at risk of geographical or occupational exposure, and also for aid workers and other professionals who may be called into filovirus endemic regions. Importantly preliminary data from phase 1 clinical trials with Ad26.ZEBOV/MVA_BN-Filo have shown a favorable vaccine safety profile [24]. This also provides an acceptable vaccine platform at commercial scale given the high production yields obtained. Small scale clinical trials in endemic areas have shown good compliance for treatment and follow-up [30], but wider immunization programs pose greater challenges for boost immunization regimens. While the field of filovirus vaccines has seen the recent development of a number of promising vaccine platforms, one challenge is to manufacture a vaccine suited to the tropical and subtropical regions where filovirus infections occur, in terms of stability, storage conditions, quantities, and transport logistics.

The results from our studies, combined with clinical data, indicate that a prophylactic multivalent filovirus vaccine is a realistic goal. Further studies in NHP and in the field are necessary to confirm that the Ad26/MVA multivalent vaccine is immunogenic in humans and can also provide protection from infection with different filoviruses.

## Disclaimer

The opinions, interpretations, conclusions, and recommendations contained here are those of the authors and are not necessarily endorsed by the US Department of Defense.

## Supporting information

S1 Fig Immunogenicity of low dose and high dose tetravalent vaccines given in a heterologous prime-boost regimen. Immunogenicity of tetravalent vaccines used in NHP study presented in Fig 2A–2D. (A) Cellular immune response over time using IFNγ ELISpot after stimulation with the indicated filovirus GP peptide pools. (B) Humoral immune response over time measured by ELISA for EBOV GP, SUDV GP and TAFV GP. The black dotted line represents the lower limit of detection.
(TIF)

S2 Fig Immunogenicity of trivalent vaccines given in a homologous or heterologous prime-boost regimen. Immunogenicity of tetravalent vaccines used in NHP study presented in Fig 3. (A) Cellular immune response over time using IFNγ ELISpot after stimulation with the indicated filovirus GP peptide pools. (B) Humoral immune response over time measured by ELISA for EBOV GP and MARV GP. The black dotted line represents the lower limit of detection.
(TIF)

S3 Fig Immunogenicity of trivalent vaccine and monovalent vaccine given in a heterologous prime-boost regimen. Immunogenicity of trivalent and monovalent vaccines used in NHP study presented in Fig 4A–4D. (A) Cellular immune response over time using IFNγ ELISpot after stimulation with the indicated filovirus GP peptide pools. (B) Humoral immune response over time measured by ELISA for MARV GP, SUDV GP and EBOV GP. The black dotted line represents the lower limit of detection.
(TIF)

S4 Fig Immunogenicity of trivalent and tetravalent vaccines given in a homologous or heterologous prime-boost regimen. Immunogenicity of trivalent and tetravalent vaccines used in NHP study presented in Fig 4E–4H. (A) Cellular immune response over time using IFNγ ELISpot after stimulation with the indicated filovirus GP peptide pools. (B) Humoral immune response over time measured by ELISA for EBOV GP, SUDV GP, TAFV GP and MARV GP. The black dotted line represents the lower limit of detection.
(TIF)

S5 Fig Immunogenicity of trivalent vaccines given in heterologous prime-boost regimen with adenovirus and MVA vectors. Immunogenicity of trivalent vaccines used in NHP study presented in Fig 5. (A) Cellular immune response over time using IFNγ ELISpot after stimulation with the indicated filovirus GP peptide pools. (B) Humoral immune response and neutralizing antibody response over time for SUDV GP and MARV GP. Horizontal dotted line represents the lower limit of detection. Vertical dotted lines indicate the time of boost immunization.
(TIF)

S1 Table Summary of study designs. (DOCX)

S2 Table Clinical parameters from the study shown in Fig 2A–2D, MARV challenge 1000pfu. (DOCX)

S3 Table Clinical parameters from the study shown in Fig 2E–2H, MARV challenge 1000pfu. (DOCX)

S4 Table Clinical parameters from the study shown in Fig 3, challenge with SUDV 1000 pfu. (DOCX)

S5 Table Clinical parameters from study shown in Fig 4A–4D, challenge with EBOV 100 pfu. (DOCX)

S6 Table Clinical parameters from the study shown in Fig 4E–4H, EBOV challenge 1000pfu. (DOCX)

S7 Table Clinical parameters from the study shown in Fig 5, challenge with EBOV 100 pfu. (DOCX)
