# Correction: A prophylactic multivalent vaccine against different filovirus species is immunogenic and provides protection from lethal infections with *Ebolavirus* and *Marburgvirus* species in non-human primates

**DOI:** 10.1371/journal.pone.0196546

**Published:** 2018-04-24

**Authors:** Benoit Callendret, Jort Vellinga, Kerstin Wunderlich, Ariane Rodriguez, Robin Steigerwald, Ulrike Dirmeier, Cedric Cheminay, Ariane Volkmann, Trevor Brasel, Ricardo Carrion, Luis D. Giavedoni, Jean L. Patterson, Chad E. Mire, Thomas W. Geisbert, Jay W. Hooper, Mo Weijtens, Jutta Hartkoorn-Pasma, Jerome Custers, Maria Grazia Pau, Hanneke Schuitemaker, Roland Zahn

The following information is missing from the Funding section: This work was supported by the National Institute of Allergy and Infectious Diseases (grant no. HHSN272200800056C to BC).
