# Supplementary figures and images for: A prophylactic multivalent vaccine against different filovirus species is immunogenic and provides protection from lethal infections with Ebolavirus and Marburgvirus species in non-human primates
Source: PLoS One. 2018 Feb 20;13(2):e0192312. doi: 10.1371/journal.pone.0192312 (PMC5819775; doi:10.1371/journal.pone.0192312)

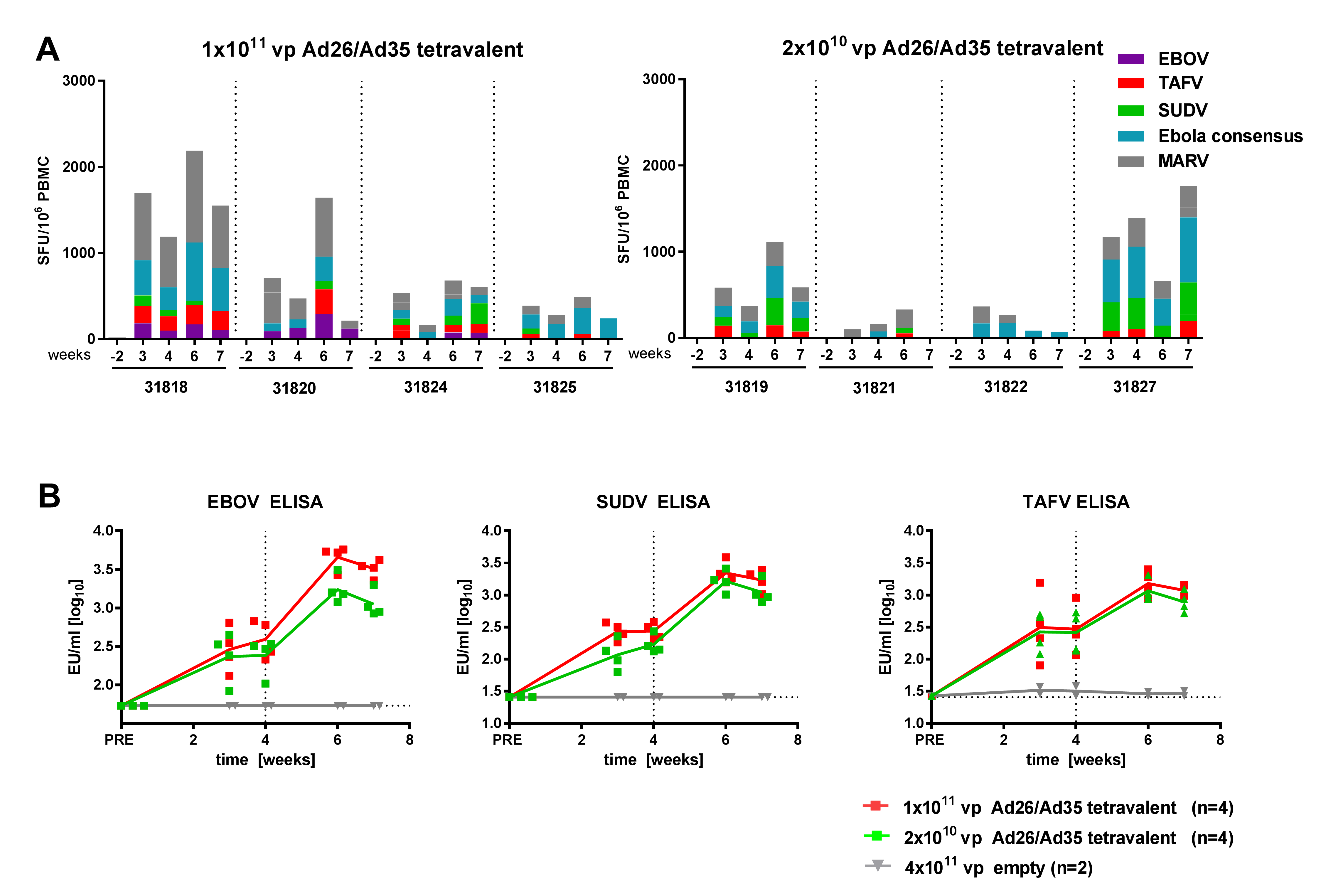

Supplement: S1 Fig — Immunogenicity of tetravalent vaccines used in NHP study presented in Fig 2A–2D. (A) Cellular immune response over time using IFNγ ELISpot after stimulation with the indicated filovirus GP peptide pools. (B) Humoral immune response over time measured by ELISA for EBOV GP, SUDV GP and TAFV GP. The black dotted line represents the lower limit of detection. (TIF) [file pone.0192312.s001.tif]

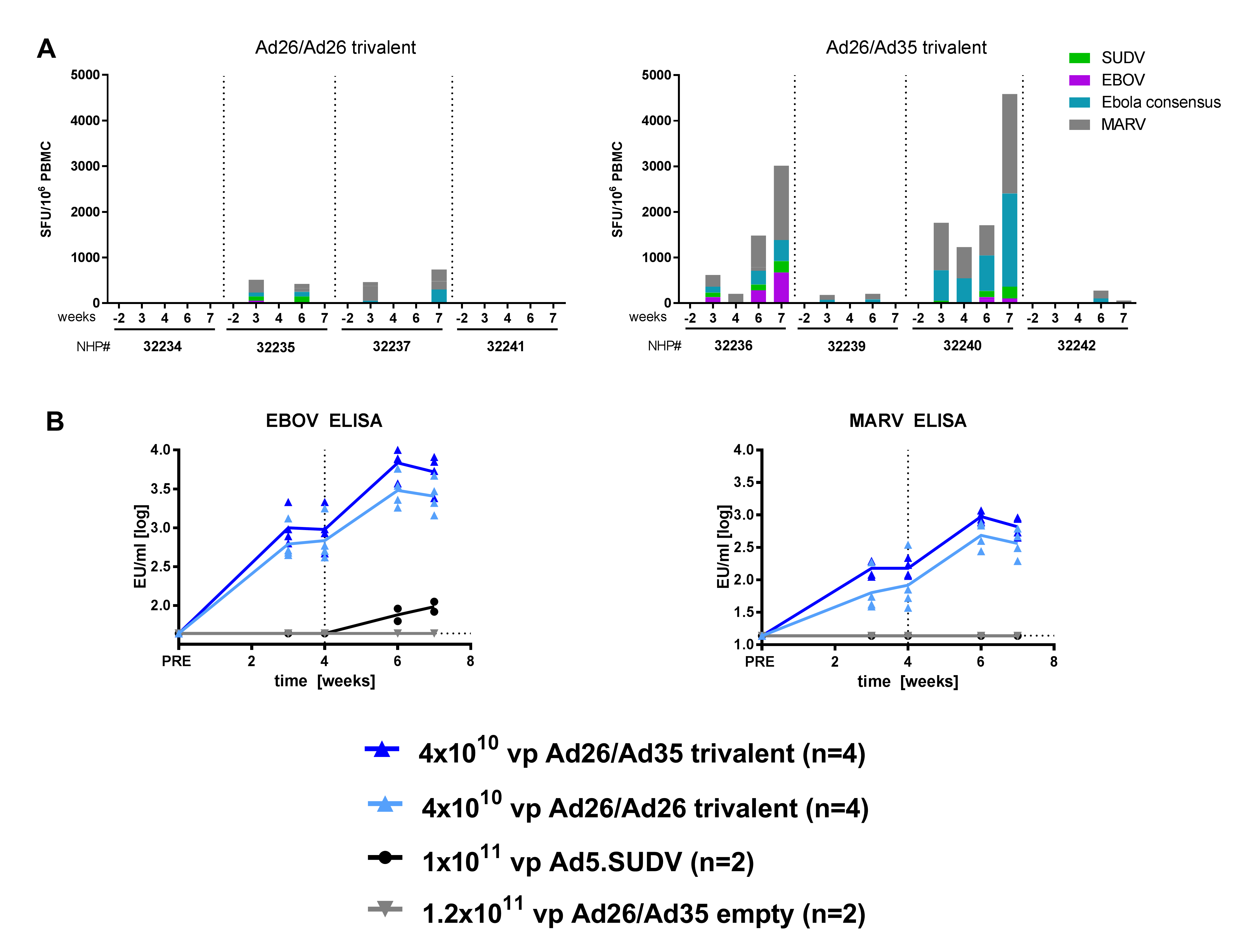

Supplement: S2 Fig — Immunogenicity of tetravalent vaccines used in NHP study presented in Fig 3. (A) Cellular immune response over time using IFNγ ELISpot after stimulation with the indicated filovirus GP peptide pools. (B) Humoral immune response over time measured by ELISA for EBOV GP and MARV GP. The black dotted line represents the lower limit of detection. (TIF) [file pone.0192312.s002.tif]

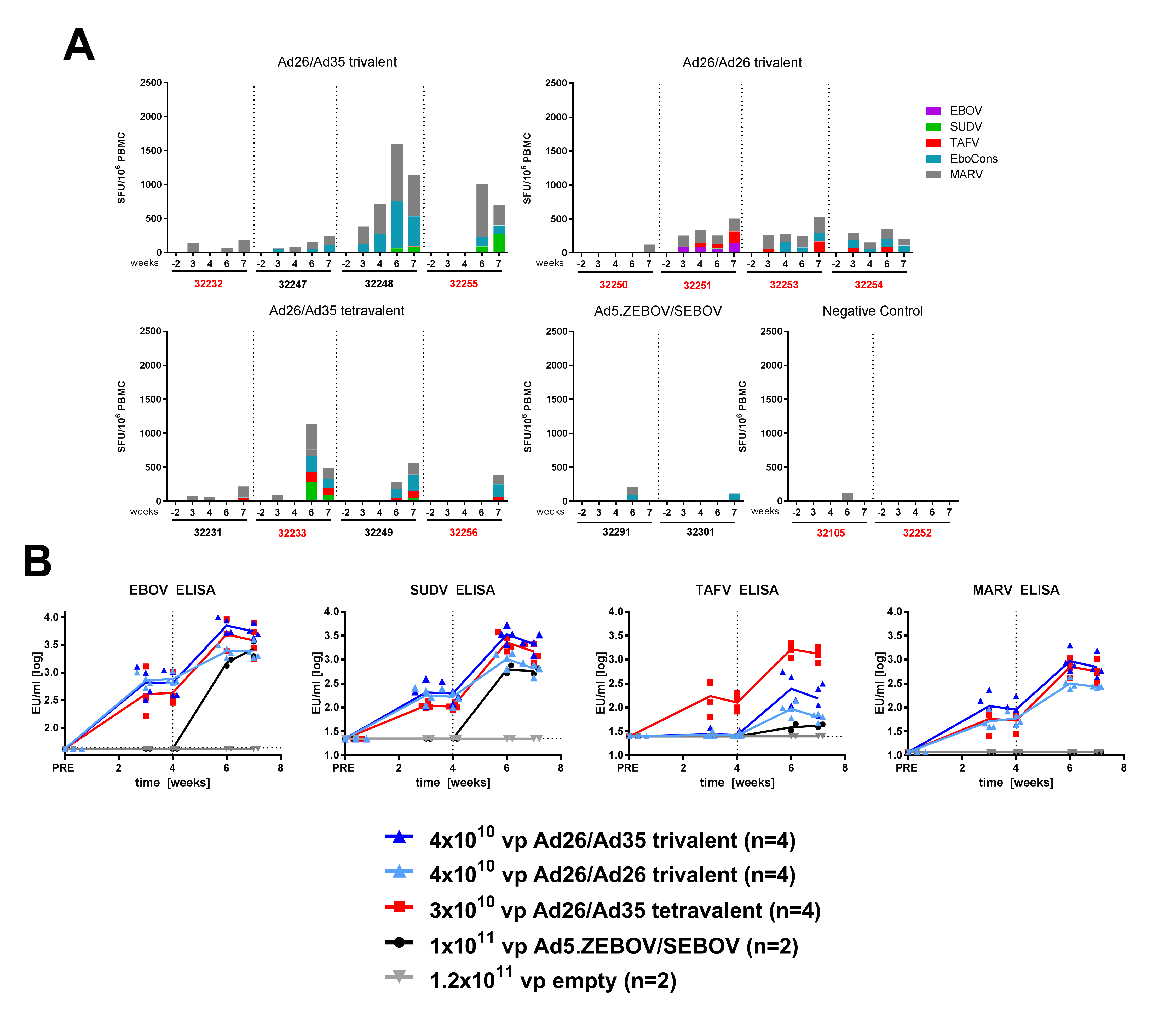

Supplement: S4 Fig — Immunogenicity of trivalent and tetravalent vaccines used in NHP study presented in Fig 4E–4H. (A) Cellular immune response over time using IFNγ ELISpot after stimulation with the indicated filovirus GP peptide pools. (B) Humoral immune response over time measured by ELISA for EBOV GP, SUDV GP, TAFV GP and MARV GP. The black dotted line represents the lower limit of detection. (TIF) [file pone.0192312.s004.tif]

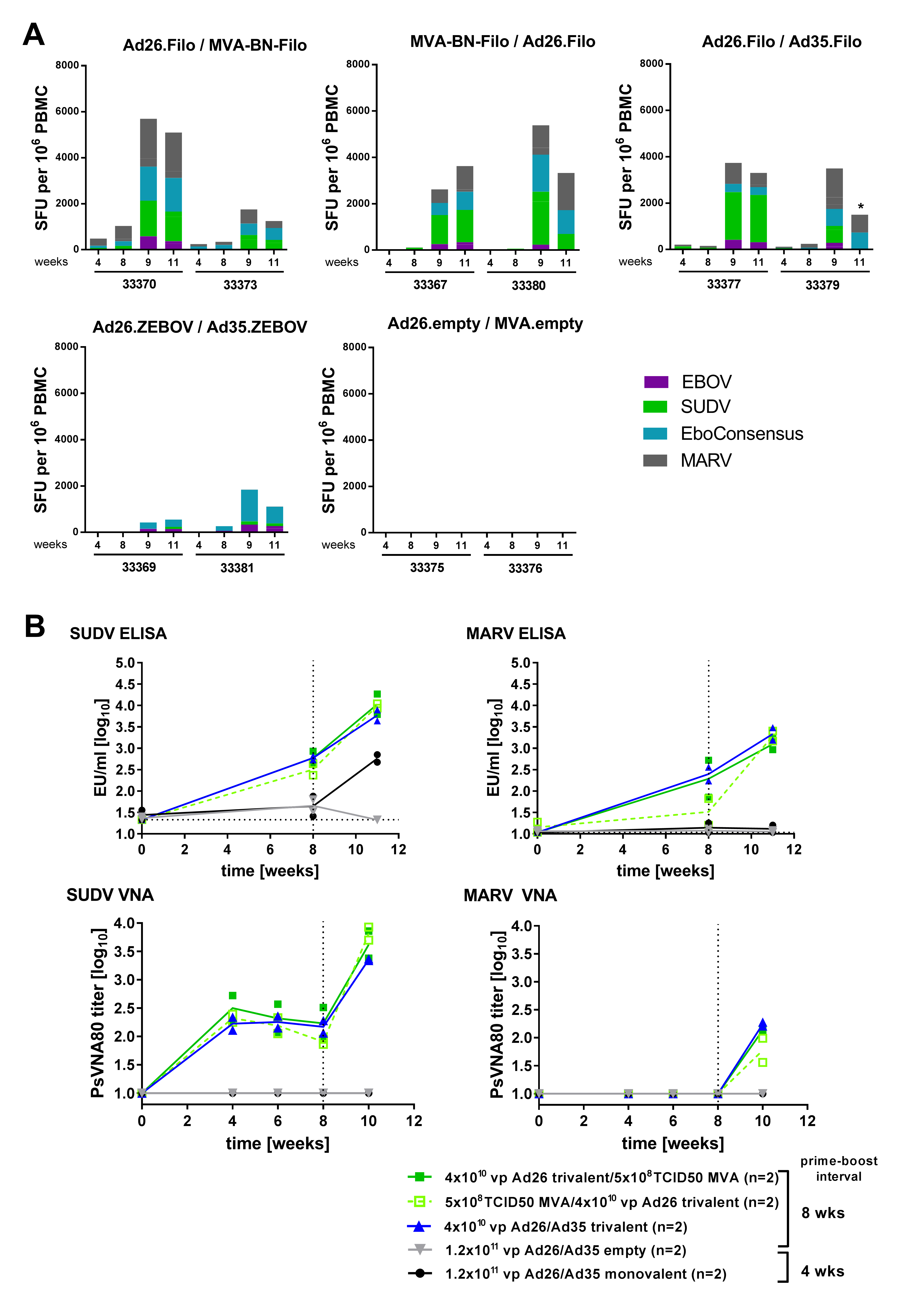

Supplement: S5 Fig — Immunogenicity of trivalent vaccines used in NHP study presented in Fig 5. (A) Cellular immune response over time using IFNγ ELISpot after stimulation with the indicated filovirus GP peptide pools. (B) Humoral immune response and neutralizing antibody response over time for SUDV GP and MARV GP. Horizontal dotted line represents the lower limit of detection. Vertical dotted lines indicate the time of boost immunization. (TIF) [file pone.0192312.s005.tif]
