# Supplementary material for: A prophylactic multivalent vaccine against different filovirus species is immunogenic and provides protection from lethal infections with Ebolavirus and Marburgvirus species in non-human primates
Source: PLoS One. 2018 Feb 20;13(2):e0192312. doi: 10.1371/journal.pone.0192312 (PMC5819775; doi:10.1371/journal.pone.0192312)
Supplement: S1 Table — (DOCX) [file pone.0192312.s006.docx]

S1 Table: Study designs

|  | **N** | **Prime**  **(Dose vp per vector)** | **Boost**  **(Dose vp per vector)** | **Imm Sched**  **(Weeks)** | **Challenge** | |
| --- | --- | --- | --- | --- | --- | --- |
|  |  |  |  |  | **Time**  **(Weeks)** | **Dose** |
| **Figure 1** | 12 | Ad26 tetravalent  (2x10^10^ vp) | Ad35 tetravalent  (2x10^10^ vp) | 0, 4 | not applicable |  |
|  | 2 | Ad26 Empty  (8x10^10^ vp) | Ad35 Empty  (8x10^10^ vp) | 0, 4 |  |  |
|  | | | | | | |
| **Figure 2 (A-D)** | 4 | Ad26 tetravalent  (1x10^11^ vp) | Ad35 tetravalent  (1x10^11^ vp) | 0, 4 | 8 | MARV  (1000 pfu) |
|  | 4 | Ad26 tetravalent  (2x10^10^ vp) | Ad35 tetravalent  (2x10^10^ vp) | 0, 4 |  |  |
|  | 2 | Ad26 MARV GP  (1x10^11^ vp) | Ad35 MARV GP  (1x10^11^ vp) | 0, 4 |  |  |
|  | 2 | Ad26 Empty  (1x10^11^ vp) | Ad35 Empty  (1x10^11^ vp) | 0, 4 |  |  |
|  | | | | | | |
| **Figure 2 (E-H)** | 3 | Ad26 trivalent  (4x10^10^ vp) | Ad26 trivalent  (4x10^10^ vp) | 0, 4 | 8 | MARV  (1000 pfu) |
|  | 1 |  | Ad5.MARV  (1x10^11^ vp) | 4 |  |  |
|  | 2 | Ad26 Empty  (1.2x10^11^ vp) | Ad26 Empty  (1.2x10^11^ vp) | 0, 4 |  |  |
|  | | | | | | |
| **Figure 3** | 4 | Ad26 trivalent  (4x10^10^ vp) | Ad35 trivalent  (4x10^10^ vp) | 0, 4 | 8 | SUDV  (1000 pfu) |
|  | 4 | Ad26 trivalent  (4x10^10^ vp) | Ad26 trivalent  (4x10^10^ vp) | 0, 4 |  |  |
|  | 2 |  | Ad5 SUDV GP  (1x10^11^ vp) | 4 |  |  |
|  | 2 | Ad26 Empty  (1.2x10^11^ vp) | Ad35 Empty  (1.2x10^11^ vp) | 0, 4 |  |  |
|  | | | | | | |
| **Figure 4 (A-D)** | 4 | Ad26 EBOV GP  (1.2x10^11^ vp) | Ad35 EBOV GP  (1.2x10^11^ vp) | 0, 4 | 8 | EBOV  (100 pfu) |
|  | 4 | Ad26 EBOV GP  (4x10^10^ vp) | Ad35 EBOV GP  (4x10^10^ vp) | 0, 4 |  |  |
|  | 4 | Ad26 trivalent  (4x10^10^ vp) | Ad35 trivalent  (4x10^10^ vp) | 0, 4 |  |  |
|  | 4 | Ad26 Empty  (1.2x10^11^ vp) | Ad35 Empty  (1.2x10^11^ vp) | 0, 4 |  |  |
|  | | | | | | |
| **Figure 4 (E-H)** | 4 | Ad26 trivalent  (4x10^10^ vp) | Ad35 trivalent  (4x10^10^ vp) | 0, 4 | 8 | EBOV  (1000 pfu) |
|  | 4 | Ad26 trivalent  (4x10^10^ vp) | Ad26 trivalent  (4x10^10^ vp) | 0, 4 |  |  |
|  | 4 | Ad26 tetravalent  (3x10^10^ vp) | Ad35 tetravalent  (3x10^10^ vp) | 0, 4 |  |  |
|  | 2 |  | Ad5 EBOV GP +SUDV GP  (1x10^11^ vp) | 4 |  |  |
|  | 2 | Ad26 Empty  (3x10^10^ vp) | Ad35 Empty  (3x10^10^ vp) | 0, 4 |  |  |
|  | | | | | | |
| **Figure 5** | 2 | Ad26 trivalent  (4x10^10^ vp) | MVA-BN-Filo  (5x10^8^ TCID50) | 0, 8 | 12 | EBOV (100 pfu) |
|  | 2 | MVA-BN-Filo  (5x10^8^ TCID50) | Ad26 trivalent  (4x10^10^ vp) | 0, 8 |  |  |
|  | 2 | Ad26 trivalent  (4x10^10^ vp) | Ad35 trivalent  (4x10^10^ vp) | 0, 8 |  |  |
|  | 2 | Ad26 Empty  (1.2x10^11^ vp) | Ad35 Empty  (1.2x10^11^ vp) | 0, 8 |  |  |
|  | 2 | Ad26 EBOV GP  (1.2x10^11^ vp) | Ad35 EBOV GP  (1.2x10^11^ vp) | 4, 8 |  |  |
