# Supplementary material for: A prophylactic multivalent vaccine against different filovirus species is immunogenic and provides protection from lethal infections with Ebolavirus and Marburgvirus species in non-human primates
Source: PLoS One. 2018 Feb 20;13(2):e0192312. doi: 10.1371/journal.pone.0192312 (PMC5819775; doi:10.1371/journal.pone.0192312)
Supplement: S2 Table — (DOCX) [file pone.0192312.s007.docx]

S2 Table: Clinical parameters from the study shown in Fig 2A-D, MARV challenge 1000pfu

| **Treatment group** | | **NHP number** | | **Day of death** | **Viral**  **load^1^** | | **Petechial**  **rash** | **Change from baseline Day 0^2^** | | | | | | | | | |
| --- | --- | --- | --- | --- | --- | --- | --- | --- | --- | --- | --- | --- | --- | --- | --- | --- | --- |
|  |  |  |  |  |  |  |  | **Temp.** | **ALT** | | | **Granulocytes** | | **PT** | | **aPTT** | |
| **Ad26/Ad35**  **1x10^11^ vp**  **tetravalent** | | 31818 | | survived | − | | − | − | − | | | − | | ↓  (14) | | ↓  (21) | |
|  |  | 31820 | | survived | − | | − | − | − | | | ↑, ↑, ↓  (10, 14, 21) | | ↑, ↑, ↑↑, ↑↑, ↑  (5, 7, 10, 14, 21) | | ↑, ↑, ↑, ↑↑, ↑  (5, 7, 10, 14, 21) | |
|  | | 31824 | | survived | − | | − | − | − | | | ↑, ↑, ↑↑  (5, 7, 10) | | − | | − | |
|  | | 31825 | | survived | − | | − | ↓↓  (5) | − | | | ↑↑, ↑↑  (7, 21) | | ↑, ↑, ↑  (5, 21, 28) | | ↑, ↑, ↑, ↑, ↑,↑  (3, 5, 10, 14, 21, 28) | |
| **Ad26/Ad35**  **2x10^10^ vp**  **tetravalent** | | 31819 | | survived | − | | − | − | − | | | ↑, ↑, ↑↑  (7, 14, 21) | | − | | ↑  (14) | |
|  |  | 31821 | | survived | − | | − | − | − | | | − | | − | | ↑, ↑  (5, 10) | |
|  | | 31822 | | survived | − | | − | − | − | | | ↑, ↑  (7, 21) | | − | | − | |
|  | | 31827 | | 10 | +++ | | + | ↑↑, ↓↓↓  (7, 10) | ↑↑↑  (10) | | | ↑, ↑↑, ↓  (3, 5, 7) | | ↑↑  (10) | | ↑↑, ↑↑  (7, 10) | |
| **Ad26/Ad35**  **monovalent** | | 31826 | | survived | − | | − | − | − | | | ↑  (10) | | ↑, ↑, ↑  (5, 10, 21) | | n.b. | |
|  | | 31828 | | survived | − | | − | − | − | | | ↑, ↑  (5, 7) | | − | | ↑, ↑  (7, 10) | |
| **empty** | | 31823 | | 10 | 1.05x10^7^ | | + | ↑  (7) | ↑↑↑  (10) | | | ↑↑, ↑↑, ↑↑, ↑↑  (3, 5, 7, 10) | | ↑↑  (10) | | ↑↑, ↑↑  (7, 10) | |
|  | | 31829 | | 10 | +++ | | + | ↑, ↓↓↓  (7, 10) | ↑↑↑  (10) | | | ↑↑  (10) | | ↑, ↑↑  (7, 10) | | ↑, ↑↑, ↑↑  (5, 7, 10) | |
| ^1^ Viral load measured in serum, in plaque forming units (PFU)/mL, from sample taken on NHP last study day. Survivors did not have measurable viral load at any timepoint. +++ Plaques too numerous to count.  ^2^ The day of the clinical finding is shown in parentheses, days after MARV challenge. Sampling times were day 0 (baseline), 3, 5, 7, 10, 14, 21 and 28 post challenge, and on the day of euthanasia for non-survivors. Petechia was scored at least twice daily.  − Negative or no change from baseline.  Rectal temperature (Temp.), increase or decrease from baseline: ↑, ↓ >2°F, ↑↑, ↓↓ >3°F, ↑↑↑, ↓↓↓ >4°F. Alanine aminotransferase (ALT), fold increase from baseline: ↑ 2 to 3 fold, ↑↑ 4 to 5 fold, ↑↑↑ 6 fold or more. Granulocyte counts, percentage change from baseline: ↑, ↓ 50%-100%, ↑↑ 101%+. Prothrombin time (PT), percentage change from baseline: ↑, ↓ 30%-49%, ↓↓,↑↑ 50%+. Activated partial thromboplastin time (aPTT), percentage change from baseline: ↑, ↓ 30%-49%, ↓↓,↑↑ 50%+ | | | | | | | | | | | | | | | | | |
| **Ad26/Ad35**  **trivalent** | 32971 | | 10 | | | 1.80x10^7^ | + | ↑, ↓↓↓  (7, 10) | | ↑↑↑  (10) | ↑↑, ↑↑  (7, 10) | | ↑↑  (10) | | ↑↑  (10) | |  |
|  | 32978 | | 10 | | | 1.34x10^7^ | + | ↑↑↑  (10) | | ↑↑↑  (10) | ↑↑, ↓  (7, 10) | | ↑↑  (10) | | ↑↑  (10) | |  |
|  | 32968 | | survived | | | − | − | ↑, ↑  (4,7) | | − | ↑↑, ↓  (7, 25) | | − | | − | |  |
|  | 32980 | | survived | | | − | − | − | | − | ↑↑, ↑↑  (7, 10) | | − | | − | |  |
| **empty** | 32965 | | 7 | | | +++ | + | − | | ↑↑↑  (7) | ↑↑, ↑↑  (4, 7) | | ↑↑  (7) | | ↑↑  (7) | |  |
|  | 32966 | | 6 | | | 1.91x10^5^ | + | ↓↓↓  (6) | | ↑↑↑  (6) | ↑↑, ↑↑  (4, 6) | | ↑  (6) | | ↑  (6) | |  |
|  | 32969 | | 9 | | | 2.88x10^4^ | + | ↓↓↓  (9) | | ↑↑, ↑↑↑  (7,9) | ↑↑, ↑↑, ↑↑  (4, 7, 9) | | − | | ↑, ↑  (7, 9) | |  |
|  | 32974 | | 9 | | | 4.38x10^6^ | + | − | | ↑  (7) | ↑, ↓  (7, 9) | | ↑↑  (9) | | ↑, ↑↑, ↑↑  (4, 7, 9) | |  |
| ^1^ Viral load measured in serum, in plaque forming units (PFU)/mL, from sample taken on NHP last study day. ^a^ viral load was detected in internal organs. Survivors did not have measurable viral load at any timepoint. +++ Plaques too numerous to count.  ^2^ The day of the clinical finding is shown in parentheses, days after EBOV challenge. Sampling times were day 0 (baseline), 4, 7, 10, 14, 21 and 28 post challenge, and on the day of euthanasia for non-survivors. Petechia was scored at least twice daily.  − Negative or no change from baseline.  Rectal temperature, increase or decrease from baseline: ↑, ↓ >2°F, ↑↑, ↓↓ >3°F, ↑↑↑, ↓↓↓ >4°F. Alanine aminotransferase (ALT), fold increase from baseline: ↑ 2 to 3 fold, ↑↑ 4 to 5 fold, ↑↑↑ 6 fold or more. Granulocyte counts, percentage change from baseline: ↑, ↓ 50%-100%, ↑↑ 101%+. Prothrombin time (PT), percentage change from baseline: ↑, ↓ 30%-49%, ↓↓,↑↑ 50%+. Activated partial thromboplastin time (aPTT), percentage change from baseline: ↑, ↓ 30%-49%, ↓↓,↑↑ 50%+. n.d. = not done | | | | | | | | | | | | | | | | |  |
