# Supplementary material for: A prophylactic multivalent vaccine against different filovirus species is immunogenic and provides protection from lethal infections with Ebolavirus and Marburgvirus species in non-human primates
Source: PLoS One. 2018 Feb 20;13(2):e0192312. doi: 10.1371/journal.pone.0192312 (PMC5819775; doi:10.1371/journal.pone.0192312)
Supplement: S3 Table — (DOCX) [file pone.0192312.s008.docx]

S3 Table: Clinical parameters from the study shown in Fig 2E-H, MARV challenge 1000pfu

| **Treatment group** | **NHP number** | **Day of death** | **Change from baseline Day 0^1^** | | |
| --- | --- | --- | --- | --- | --- |
|  |  |  | **Temp.** | **PT** | **aPTT** |
| **Ad26/Ad35**  **tetravalent** | 32246 | survived | ↑  (21) | ↓  (5) | ↓  (21) |
|  | 32289 | survived | − | − | − |
|  | 32290 | survived | − | − | − |
| **Ad5.MARV** | 32436 | survived | ↓, ↓↓  (5, 9) | − | − |
| **empty** | 32288 | 9 | − | ↑↑, ↑↑  (7, 9) | ↑↑, ↑↑  (7, 9) |
|  | 32438 | 9 | − | ↑  (7) | ↓  (7) |
| ^1^ The day of the clinical finding is shown in parentheses, days after MARV challenge. Sampling times were day 0 (baseline), 5, 7, 9, 14, 21 and 28 post challenge, and on the day of euthanasia for non-survivors.  − Negative or no change from baseline.  Rectal temperature (Temp.), increase or decrease from baseline: ↑, ↓ >2°F, ↑↑, ↓↓ >3°F, ↑↑↑, ↓↓↓ >4°F  Prothrombin time (PT), percentage change from baseline: ↑, ↓ 30%-49%, ↓↓,↑↑ 50%+  Activated partial thromboplastin time (aPTT), percentage change from baseline: ↑, ↓ 30%-49%, ↓↓,↑↑ 50%+ | | | | | |
