# Supplementary material for: A prophylactic multivalent vaccine against different filovirus species is immunogenic and provides protection from lethal infections with Ebolavirus and Marburgvirus species in non-human primates
Source: PLoS One. 2018 Feb 20;13(2):e0192312. doi: 10.1371/journal.pone.0192312 (PMC5819775; doi:10.1371/journal.pone.0192312)
Supplement: S4 Table — (DOCX) [file pone.0192312.s009.docx]

S4 Table: Clinical parameters from the study shown in Fig 3, challenge with SUDV 1000 pfu

| **Treatment group** | **NHP number** | **Day of death** | **Viral load^1^** | **Petechial rash** | **Change from baseline Day 0^2^** | | | | |
| --- | --- | --- | --- | --- | --- | --- | --- | --- | --- |
|  |  |  |  |  | **Temperature** | **ALT** | **Granulocytes** | **PT** | **aPTT** |
| **Ad26/Ad35 trivalent** | 32239 | survived | − | − | ↓  (7) | ↓  (28) | ↑↑, ↑, ↑↑, ↑↑, ↑↑, ↑↑  (3, 5, 7, 10, 14, 21) | − | ↓  (7) |
|  | 32236 | survived | − | − | − | ↓  (29) | ↓, ↑↑,↑  (3, 7, 29) | − | − |
|  | 32240 | survived | − | − | ↑↑  (21) | − | ↓, ↑↑  (5, 7) | − | − |
|  | 32242 | 9 | −^a^ | + | ↑↑, ↑↑↑, ↑↑  (5, 7, 9) | ↑,↑↑  (7, 9) | ↑↑, ↑↑, ↑↑  (3, 5, 7) | − | ↑↑,↑↑  (7, 9) |
| **Ad26/Ad26 trivalent** | 32237 | survived | − | − | − | − | ↑↑, ↑, ↑↑  (7, 10, 29) | ↓, ↓  (10, 21) | ↓↓  (21) |
|  | 32235 | survived | − | − | ↓↓↓  (5) | − | ↑  (21) | − | ↑↑  (5) |
|  | 32234 | survived | − | − | ↑  (7) | − | ↑↑, ↑↑, ↑↑, ↑, ↑↑  (3, 5, 7, 10, 14) | ↓↓  (7) | − |
|  | 32241 | 8 | 2.18x10^7^ | + | ↑↑, ↑  (5, 7) | ↓, ↑  (5, 8) | ↑↑,↑↑  (3, 5) | ↑↑  (8) | ↑↑,↑↑  (7, 8) |
| **Ad5.SUDV** | 32244 | survived | − | − | − | − | ↑↑, ↑↑, ↑↑, ↑↑  (5, 7, 21, 28) | − | ↓, ↓↓  (10, 21) |
|  | 32300 | 10 | −^a^ | − | ↑↑, ↑↑↑, ↑↑  (5, 7, 10) | ↓, ↑  (7, 10) | ↑↑, ↑↑  (5, 7) | − | ↑  (10) |
| **empty** | 32245 | 8 | 1.35x10^7^ | + | ↑, ↑↑↑, ↑↑↑  (5, 7, 8) | ↑  (6) | ↑↑, ↑↑, ↑↑  (3, 5, 7) | ↑, ↑↑, ↑  (5, 7, 8) | ↑, ↑↑, ↑↑, ↑↑  (3, 5, 7, 8) |
|  | 32243 | 6 | 3.08x10^6^ | + | ↑↑↑  (6) | − | ↑↑, ↑↑  (3, 5) | ↑↑  (5) | ↑↑  (5) |
| ^1^ Viral load measured in serum, in plaque forming units (PFU)/mL, from sample taken on NHP last study day. ^a^ viral load was detected in internal organs. Survivors did not have measurable viral load at any timepoint.  ^2^ The day of the clinical finding is shown in parentheses, days after SUDV challenge. Sampling times were day 0 (baseline), 3, 5, 7, 10, 14 and 28 post challenge, and on the day of euthanasia for non-survivors. Petechia was scored at least twice daily.  − Negative or no change from baseline.  Rectal temperature, increase or decrease from baseline: ↑, ↓ >2°F, ↑↑, ↓↓ >3°F, ↑↑↑, ↓↓↓ >4°F. Alanine aminotransferase (ALT), fold increase from baseline: ↑ 2 to 3 fold, ↑↑ 4 to 5 fold, ↑↑↑ 6 fold or more. Granulocyte counts, percentage change from baseline: ↑, ↓ 50%-100%, ↑↑ 101%+. Prothrombin time (PT), percentage change from baseline: ↑, ↓ 30%-49%, ↓↓,↑↑ 50%+. Activated partial thromboplastin time (aPTT), percentage change from baseline: ↑, ↓ 30%-49%, ↓↓,↑↑ 50%+ | | | | | | | | | |
