# Supplementary material for: A prophylactic multivalent vaccine against different filovirus species is immunogenic and provides protection from lethal infections with Ebolavirus and Marburgvirus species in non-human primates
Source: PLoS One. 2018 Feb 20;13(2):e0192312. doi: 10.1371/journal.pone.0192312 (PMC5819775; doi:10.1371/journal.pone.0192312)
Supplement: S5 Table — (DOCX) [file pone.0192312.s010.docx]

S5 Table: Clinical parameters from study shown in Fig 4A-D, challenge with EBOV 100 pfu

| **Treatment group** | **NHP number** | **Day of death** | **Viral load^1^** | **Petechial rash** | **Change from baseline Day 0^2^** | | | | |
| --- | --- | --- | --- | --- | --- | --- | --- | --- | --- |
|  |  |  |  |  | **Temperature** | **ALT** | **Granulocytes** | **PT** | **aPTT** |
| **Ad26/Ad35**  **monovalent**  **1.2x10^11^** | 32975 | 9 | −^a^ | + | ↑  (7) | − | ↑↑  (7) | − | − |
|  | 32963 | survived | − | − | − | − | ↑  (10) | − | − |
|  | 32967 | survived | − | − | − | − | ↓  (7) | − | − |
|  | 32972 | survived | − | − | − | − | − | − | − |
| **Ad26/Ad35**  **monovalent**  **4x10^10^** | 32433 | 9 | −^a^ | − | − | − | ↑↑  (7) | − | ↑↑  (4) |
|  | 32976 | 10 | 1.80x10^6^ | + | ↑↑  (7) | ↑↑↑  (10) | ↑↑, ↑↑  (7, 10) | ↑↑  (10) | ↑↑  (10) |
|  | 32973 | survived | − | − | − | − | ↓, ↓, ↓, ↓, ↓, ↓  (4,7,10,14,21,28) | − | ↑, ↑, ↑, ↑  (4, 7, 10, 14) |
|  | 32979 | survived | − | − | − | − | − | − | ↓  (7) |
| **Ad26/Ad35**  **trivalent** | 32971 | 10 | 1.80x10^7^ | + | ↑, ↓↓↓  (7, 10) | ↑↑↑  (10) | ↑↑, ↑↑  (7, 10) | ↑↑  (10) | ↑↑  (10) |
|  | 32978 | 10 | 1.34x10^7^ | + | ↑↑↑  (10) | ↑↑↑  (10) | ↑↑, ↓  (7, 10) | ↑↑  (10) | ↑↑  (10) |
|  | 32968 | survived | − | − | ↑, ↑  (4,7) | − | ↑↑, ↓  (7, 25) | − | − |
|  | 32980 | survived | − | − | − | − | ↑↑, ↑↑  (7, 10) | − | − |
| **empty** | 32965 | 7 | +++ | + | − | ↑↑↑  (7) | ↑↑, ↑↑  (4, 7) | ↑↑  (7) | ↑↑  (7) |
|  | 32966 | 6 | 1.91x10^5^ | + | ↓↓↓  (6) | ↑↑↑  (6) | ↑↑, ↑↑  (4, 6) | ↑  (6) | ↑  (6) |
|  | 32969 | 9 | 2.88x10^4^ | + | ↓↓↓  (9) | ↑↑, ↑↑↑  (7,9) | ↑↑, ↑↑, ↑↑  (4, 7, 9) | − | ↑, ↑  (7, 9) |
|  | 32974 | 9 | 4.38x10^6^ | + | − | ↑  (7) | ↑, ↓  (7, 9) | ↑↑  (9) | ↑, ↑↑, ↑↑  (4, 7, 9) |
| ^1^ Viral load measured in serum, in plaque forming units (PFU)/mL, from sample taken on NHP last study day. ^a^ viral load was detected in internal organs. Survivors did not have measurable viral load at any timepoint. +++ Plaques too numerous to count.  ^2^ The day of the clinical finding is shown in parentheses, days after EBOV challenge. Sampling times were day 0 (baseline), 4, 7, 10, 14, 21 and 28 post challenge, and on the day of euthanasia for non-survivors. Petechia was scored at least twice daily.  − Negative or no change from baseline.  Rectal temperature, increase or decrease from baseline: ↑, ↓ >2°F, ↑↑, ↓↓ >3°F, ↑↑↑, ↓↓↓ >4°F. Alanine aminotransferase (ALT), fold increase from baseline: ↑ 2 to 3 fold, ↑↑ 4 to 5 fold, ↑↑↑ 6 fold or more. Granulocyte counts, percentage change from baseline: ↑, ↓ 50%-100%, ↑↑ 101%+. Prothrombin time (PT), percentage change from baseline: ↑, ↓ 30%-49%, ↓↓,↑↑ 50%+. Activated partial thromboplastin time (aPTT), percentage change from baseline: ↑, ↓ 30%-49%, ↓↓,↑↑ 50%+. n.d. = not done | | | | | | | | | |
