# Supplementary material for: A prophylactic multivalent vaccine against different filovirus species is immunogenic and provides protection from lethal infections with Ebolavirus and Marburgvirus species in non-human primates
Source: PLoS One. 2018 Feb 20;13(2):e0192312. doi: 10.1371/journal.pone.0192312 (PMC5819775; doi:10.1371/journal.pone.0192312)
Supplement: S6 Table — (DOCX) [file pone.0192312.s011.docx]

S6 Table: Clinical parameters from the study shown in Fig 4E-H, EBOV challenge 1000pfu

| **Treatment group** | **NHP number** | **Day of death** | **Viral**  **load^1^** | **Petechial**  **rash** | **Change from baseline Day 0^2^** | | |
| --- | --- | --- | --- | --- | --- | --- | --- |
|  |  |  |  |  | **Temperature** | **ALT** | **Granulocytes** |
| **Ad26/Ad35**  **trivalent** | 32232 | 7 | 4.75x10^6^ | + | ↑  (4) | ↑↑↑  (7) | ↑↑  (4) |
|  | 32255 | 7 | 1.83x10^7^ | + | ↑↑, ↑↑  (4, 7) | ↑, ↑  (4, 7) | ↑↑, ↑↑  (4, 7) |
|  | 32247 | survived | − | − | ↑  (7) | ↑↑↑, ↑  (10, 14) | ↓, ↓, ↓  (10, 21, 27) |
|  | 32248 | survived | − | − | ↓, ↑, ↓  (4, 7, 14) | − | ↑↑, ↑↑, ↓, ↓, ↓, ↓  (4, 7, 10, 14, 21, 27) |
| **Ad26/Ad26**  **trivalent** | 32250 | 7 | n.d. | + | − | − | ↑↑  (4) |
|  | 32251 | 7 | 4.25x10^7^ | + | ↑↑, ↑↑  (4, 7) | ↑↑  (7) | ↑↑, ↓  (4, 7) |
|  | 32253 | 7 | 2.65x10^7^ | + | ↑, ↑  (4, 7) | ↑  (7) | ↑↑, ↑↑  (4, 7) |
|  | 32254 | 6 | 7.50x10^7^ | + | ↑↑↑, ↓  (4, 6) | ↑↑↑  (6) | ↑↑  (4) |
| **Ad26/Ad35**  **tetravalent** | 32233 | 8 | 6.25x10^5^ | + | ↑, ↑  (7, 8) | ↑, ↑↑↑  (4, 8) | ↑↑, ↑↑  (4, 7) |
|  | 32256 | 8 | 6.00x10^7^ | + | − | ↑↑, ↑↑↑  (7, 8) | ↓  (7) |
|  | 32231 | survived | − | − | ↓  (10) | − | ↓, ↑↑, ↓↓  (4, 7, 21) |
|  | 32249 | survived | − | − | ↑, ↓  (7, 10) | − | ↑↑, ↑↑, ↓, ↓, ↑↑  (4, 7, 10, 14, 27 ) |
| **Ad5.ZEBOV/**  **SEBOV** | 32291 | survived | − | − | ↓  (10) | ↑, ↑, ↑↑↑, ↑↑, ↑  (4, 7, 10, 21) | ↑↑, ↑↑, ↑, ↑  (4, 7, 10, 14) |
|  | 32301 | survived | − | − | ↑  (7) | ↑, ↑↑, ↑  (7,10,14) | ↓, ↓, ↓  (14, 21, 27) |
| **empty** | \| 32105 \| \| --- \| \|  \| | 7 | 1.10x10^6^ | + | − | ↑  (7) | ↑↑, ↑↑  (4, 7) |
|  | 32252 | 7 | 7.00x10^6^ | + | − | ↑, ↑↑  (4, 7) | ↑↑, ↑↑  (4, 7) |
| ^1^ Viral load measured in serum, in plaque forming units (PFU)/mL, from sample taken on NHP last study day. Survivors did not have measurable viral load at any timepoint. +++ Plaques too numerous to count.  ^2^ The day of the clinical finding is shown in parentheses, days after EBOV challenge. Sampling times were day 0 (baseline), 4, 7, 10, 14, 21 and 27 or 28 post challenge, and on the day of euthanasia for non-survivors. Petechia was scored at least twice daily.  − Negative or no change from baseline.  Rectal temperature, increase or decrease from baseline: ↑, ↓ >2°F, ↑↑, ↓↓ >3°F, ↑↑↑, ↓↓↓ >4°F. Alanine aminotransferase (ALT), fold increase from baseline: ↑ 2 to 3 fold, ↑↑ 4 to 5 fold, ↑↑↑ 6 fold+. Granulocyte counts, percentage change from baseline: ↑, ↓ 50%-100%, ↑↑ 101%+. n.d.= not done | | | | | | | |
