# Supplementary material for: A prophylactic multivalent vaccine against different filovirus species is immunogenic and provides protection from lethal infections with Ebolavirus and Marburgvirus species in non-human primates
Source: PLoS One. 2018 Feb 20;13(2):e0192312. doi: 10.1371/journal.pone.0192312 (PMC5819775; doi:10.1371/journal.pone.0192312)
Supplement: S7 Table — (DOCX) [file pone.0192312.s012.docx]

S7 Table: Clinical parameters from the study shown in Fig 5, challenge with EBOV 100 pfu

| **Treatment group** | **NHP number** | **Day of death** | **Viral load^1^** | **Petechial rash** | **Change from baseline Day 0^2^** | | | | |
| --- | --- | --- | --- | --- | --- | --- | --- | --- | --- |
|  |  |  |  |  | **Temperature** | **ALT** | **Granulocytes** | **PT** | **aPTT** |
| **Ad26 trivalent**  **/MVA** | 33370 | survived | − | − | − | − | ↑,↑,↓  (3, 14, 27) | − | ↑↑  (3) |
|  | 33373 | survived | − | − | − | − | ↑  (3) | − | ↑,↑↑  (9, 27) |
| **MVA/**  **Ad26 trivalent** | 33367 | survived | − | − | − | − | − | − | ↑  (9) |
|  | 33380 | survived | − | − | − | − | ↓  (6) | − | − |
| **Ad26/Ad35**  **trivalent** | 33377 | survived | − | − | − | − | − | − | ↑  (6) |
|  | 33379 | survived | − | − | − | − | ↑,↓,↑  (3, 6, 9) | − | ↑↑,↑↑,↑  (3, 6, 9) |
| **Ad26/Ad35**  **monovalent** | 33369 | survived | − | − | ↑,↑  (3, 27) | − | ↑  (14) | ↓,↓  (6, 27) | − |
|  | 33381 | survived | − | − | − | − | ↓,↓  (6, 14) | − | − |
| **empty** | 33375 | 7 | 1.35x10^7^ | + | ↑↑↑  (6) | ↑  (6) | ↑↑  (6) | ↑↑,↑↑  (6, 7) | ↑,↑↑  (6, 7) |
|  | 33376 | 7 | 3.08x10^6^ | + | ↑↑↑, ↓↓↓  (6, 7) | − | ↑↑  (6) | ↑,↑↑  (6, 7) | ↑↑  (7) |
| ^1^ Viral load measured in serum, in plaque forming units (PFU)/mL, from sample taken on NHP last study day. Survivors did not have measurable viral load at any timepoint.  ^2^ The day of the clinical finding is shown in parentheses, days after EBOV challenge. Sampling times were day 0 (baseline), 3, 6, 9, 14, and 27 or 28 post challenge, and on the day of euthanasia for non-survivors. Petechia was scored at least twice daily.  − Negative or no change from baseline.  Rectal temperature, increase or decrease from baseline: ↑, ↓ >2°F, ↑↑, ↓↓ >3°F, ↑↑↑, ↓↓↓ >4°F  Alanine aminotransferase (ALT), fold increase from baseline: ↑ 2 to 3 fold, ↑↑ 4 to 5 fold, ↑↑↑ 6 fold or more  Granulocyte counts, percentage change from baseline: ↑, ↓ 50%-100%, ↑↑ 101%+  Prothrombin time (PT), percentage change from baseline: ↑, ↓ 30%-49%, ↓↓,↑↑ 50%+  Activated partial thromboplastin time (aPTT), percentage change from baseline: ↑, ↓ 30%-49%, ↓↓,↑↑ 50%+ | | | | | | | | | |
